# Advances in Coumarin Fluorescent Probes for Medical Diagnostics: A Review of Recent Developments

**DOI:** 10.3390/bios16010036

**Published:** 2026-01-02

**Authors:** Katarzyna Szwaczko, Aleksandra Kulkowska, Arkadiusz Matwijczuk

**Affiliations:** 1 Department of Organic Chemistry and Crystallochemistry, Institute of Chemical Sciences, Faculty of Chemistry, Marie Curie-Skłodowska University, Gliniana 33, 20-614 Lublin, Poland; kulkowskaaleksandra@gmail.com; 2 Department of Biophysics, Faculty of Environmental Biology, University of Life Sciences in Lublin, Akademicka 13, 20-950 Lublin, Poland; arkadiusz.matwijczuk@up.lublin.pl

**Keywords:** coumarin, fluorescent probes, bioimaging, NIR

## Abstract

This review summarizes recent advances (2023–2025) in coumarin-based fluorescent probes, highlighting their structural modularity, tunable VIS–NIR photophysics, and broad applicability in detecting metal ions, biothiols, ROS/RNS, organelle-specific microenvironments, and amyloid-β aggregates. Particular emphasis is placed on multifunctional and organelle-targeted probes, as well as emerging NIR-emissive and theranostic systems enabling deep-tissue imaging and modulation of pathological processes. The perspectives section outlines current limitations and future directions toward clinically relevant coumarin-based imaging tools. A though the review focuses on literature published from 2023 onward, several earlier studies are cited selectively to clarify fluorescence mechanisms, illustrate reaction pathways, or provide essential photophysical benchmarks necessary for contextual understanding.

## 1. Introduction

Fluorescence is an optical phenomenon commonly used in the design of modern biosensors and chemical probes. Due to its high sensitivity and selectivity, this technique plays a key role in medical diagnostics, cell biology, and environmental and food quality monitoring. Optical biosensors are based on the phenomenon of fluorescence. Various types of fluorescent dyes, such as quantum dots, dyes, and fluorescent proteins, can be used as biosensors [1,2,3,4,5,6,7,8,9,10,11,12,13].

Small-molecule fluorescent probes play a key role in the development of medical imaging and diagnostic methods [14,15,16,17,18,19,20,21]. Their use offers many advantages, as they are easy to synthesize and chemically modify, display high sensitivity and selectivity, and are also non-toxic. Recently, fluorescent probes have found wide application in the diagnosis and treatment of diseases, including the localization of tumors and the visualization of metabolic processes at the cell level. The dynamic development of biophysical techniques and analytical chemistry has meant that fluorescent dyes have opened up new possibilities in interdisciplinary research combining chemistry, biology, and medicine.

Among the numerous fluorophores, coumarin derivatives are particularly significant due to their favorable photophysical properties, high stability, and the possibility of simple modification of their skeleton. A properly designed coumarin molecule architecture allows control of the wavelength of the emitted light, enabling its specific diagnostic application [22,23,24,25,26].

The objective of this review is to elucidate recent advancements in the design and applications of fluorescent probes derived from the coumarin skeleton, with a specific focus on innovations developed from 2023 to 2025. While the core of this review is dedicated to the most recent literature, earlier foundational publications are selectively cited to clarify fluorescence mechanisms, illustrate reaction pathways, or provide essential photophysical benchmarks necessary to contextualize recent progress.

## 2. Coumarins, Structure, Diversity, and Synthetic Pathways

Coumarins are heterocyclic secondary metabolites of plants that are widely distributed in nature. These compounds have several attractive features, such as low molecular weight, simple structure, high bioavailability, satisfactory solubility in most organic solvents, and low toxicity. For many years, they have attracted the attention of scientists and have played a leading role in research on potential drugs, as covered in numerous review articles [27,28,29,30,31,32,33,34,35,36,37]. However, the use of coumarins is not limited to biology. Due to their attractive photophysical properties, these derivatives serve an important role in optoelectronic technologies as laser dyes, luminophores, and components of luminescent materials.

**Figure 1:**
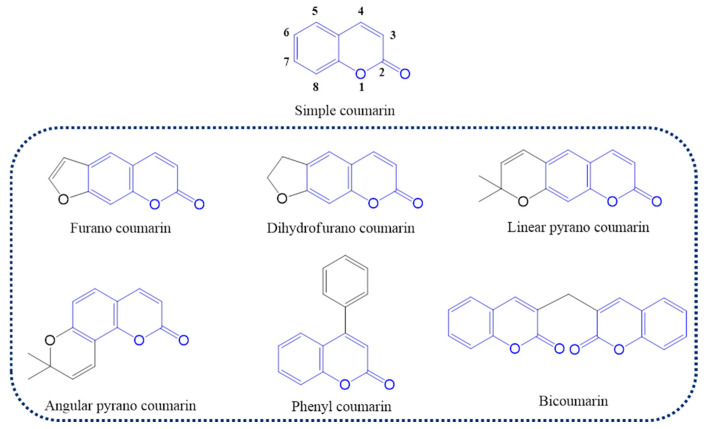
Structural classification of coumarins: simple coumarin and its main derivatives.

Coumarins are a numerous group of compounds that include both simple derivatives and more complicated condensed systems. There are six main structural classes of coumarins in the literature: simple coumarin derivatives, furanocoumarins, pyrano coumarins, dihydrofurano coumarins (with a linear or angular arrangement), phenyl coumarins, and bicoumarins (Figure 1). This diversity emphasizes the broad scope for modification of the coumarin skeleton, which is essential in the design of fluorescent probes with specific photophysical properties.

**Scheme 1:**
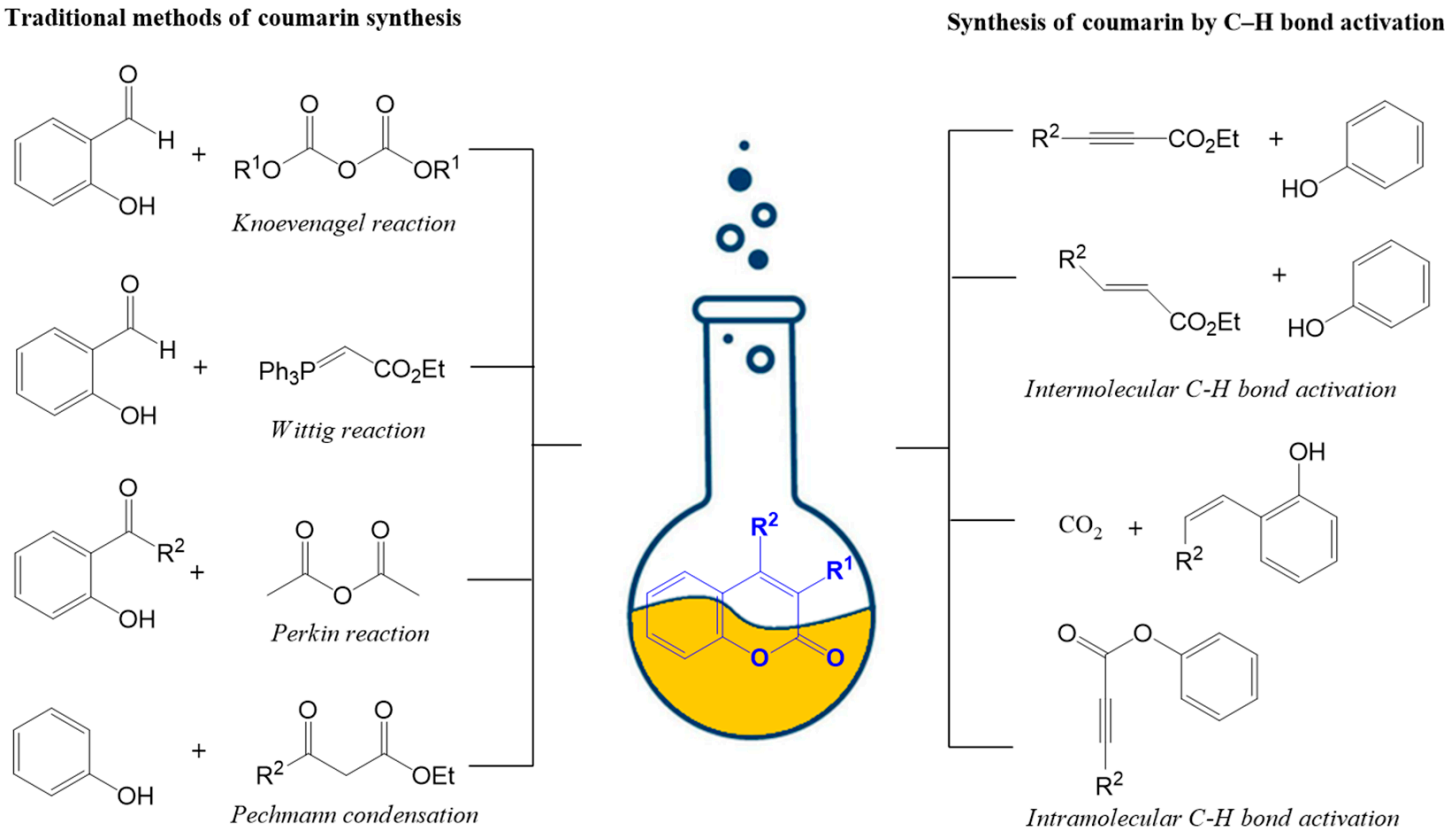
Representative routes for the synthesis of the coumarin skeleton.

The synthesis of coumarins has also been the subject of intensive research in organic chemistry for many years. There are a number of classic, simple, and effective methods for preparing coumarin derivatives, such as the Perkin reaction, von Pechmann condensation, Knoevenagel condensation, Baylis–Hillman reaction, Michael addition, and Kostanecki reaction (Scheme 1) [38,39,40,41,42,43]. Depending on the substrates and conditions used, these methods allow for the rapid synthesis of both structurally simple coumarin cores and substituted compounds, often in a single reaction step.

The chemist can precisely control the substitutions in key positions of the coumarin ring (most often 3, 4, and 7), which allows for the modulation of the electronic and optical properties of the compounds. Recently, classical condensation methods have been further complemented by modern catalytic approaches, including C–H bond activation reactions catalyzed by transition metal complexes (e.g., Pd, Ru, Co, Fe, Au) [44,45,46,47,48,49]. They allow both the construction of a coumarin skeleton de novo and the introduction of new functional groups into an already formed heterocyclic system, often under mild conditions and with strong compatibility with various functional groups.

## 3. Optical Properties and Mechanisms of Action Fluorescent Probes

**Figure 2:**
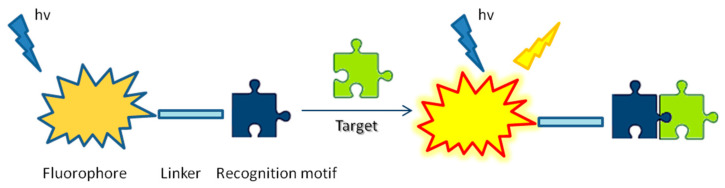
Fluorescence probe operating scheme.

Fluorescent probes are molecules that emit light when excited by an external energy source. Typical components of a fluorescent probe are a fluorescent group (fluorophore), a linker, and a target recognition motif (Figure 2). The fluorophore provides the fluorescent signal and determines the sensitivity of the fluorescent probe. The fluorophore changes its fluorescence in response to an external stimulus, such as interaction with biomolecules, reactive chemical compounds, light, or environmental changes. A properly designed probe permits biological testing and the real-time imaging of biological systems.

In designing biological probes, it is important for the fluorescent group to have appropriate photophysical properties, such as high fluorescence quantum yield (Φ), adequate photochemical stability, and a large Stokes shift (the difference between the absorption maximum λ_abs_ and the emission maximum λ_em_), which minimizes the overlap of excitation and emission signals. The position of the emission spectrum is also important, and fluorophores emitting light in the visible or NIR range ensure better tissue penetration and higher detection sensitivity. The selectivity and functionalization of fluorophores, i.e., the introduction of groups that recognize specific biomarkers, is equally important in their construction. In addition, probes should be characterized by adequate solubility, biological compatibility, chemical stability, low toxicity, and high bioavailability. Selecting the appropriate fluorophore for biological probes is, therefore, a difficult process and requires simultaneous consideration of photophysical and biological parameters to preserve cell viability and to enable clear imaging of the biological system under study.

**Figure 3:**
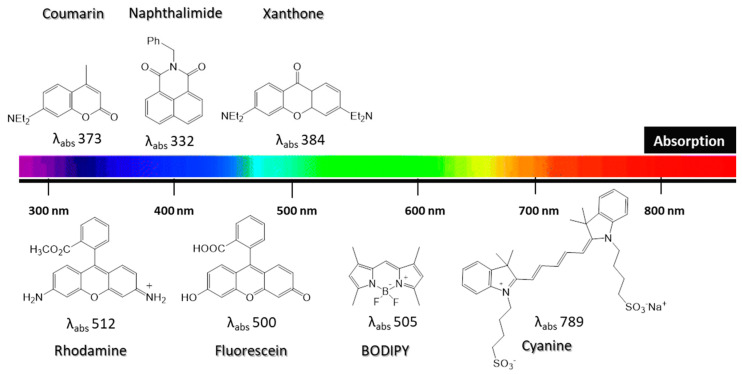
Selected fluorophores and their approximate absorption ranges.

To emit fluorescence, fluorophores are excited by light of the appropriate wavelength. Excitation in the ultraviolet range can damage tissue, while near-infrared radiation leads to cell heating and limits its use in in vivo testing. Blue and green excitation light is suitable for surface imaging, but it weakly penetrates tissue, so it works well mainly in small animals or in cell studies. Yellow and red light, on the other hand, generates autofluorescence in cells because hemoglobin and other endogenous dyes are excited in this range. Therefore, fluorophores excited by deep red or near-infrared light (so-called biological window), combining good tissue penetration with low autofluorescence, are optimal for biological applications. To date, many fluorescent probes have been constructed based on simple organic compound skeletons or their complexes with metals. Among the well-known fluorophores are coumarins, fluoresceins, rhodamines, BODIPYs (boron dipyrromethene), quinolines, naphthalimides, flavones, styrenes, and others [50,51,52,53,54,55,56]. These molecules differ in both their absorption range (Figure 3) and Stokes shift. For example, BODIPYs have high quantum yields but small Stokes shifts, while coumarins and quinolines usually have large Stokes shifts but shorter excitation wavelengths.

**Figure 4:**
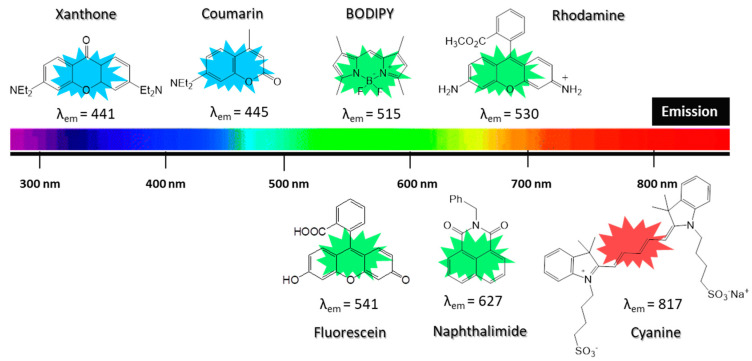
Representative emission ranges of common fluorophore scaffolds.

In imaging cells and organisms, it is preferable to emit light at wavelengths that provide high signal contrast, low autofluorescence, and deeper tissue penetration. Fluorophores emitting in the red or near-infrared range perform best because light with higher wavelengths undergoes less dispersion and absorption [57,58]. In cellular imaging (fluorescence microscopy, confocal microscopy, and spinning disk microscopy) and in vivo systems, detectors such as high-sensitivity CCD/CMOS cameras and photomultiplier tubes (PMTs) are most effective in the red and NIR ranges [59,60]. Therefore, probes emitting in these areas offer a brighter signal, less photodamage, and better contrast, which is crucial for real-time monitoring of biological processes. However, for many molecular probes (including coumarins, xanthones, quinolines, fluorescein), emission naturally occurs in the blue or green range, which, despite its shallower imaging depth, is characterized by very high brightness, low toxicity, and excellent compatibility with microscopic detection (Figure 4). In practice, the emission of these probes is fully applicable in imaging adherent cells, superficial tissues, organelles (mitochondria, ER, lysosomes), and thin animal models (e.g., *Danio rerio*, mammalian embryos). Furthermore, by appropriately designing the donor–acceptor fragments and extending the π coupling of the fluorophores, it is possible to shift their emission towards yellow and orange, which improves their application potential in biological systems.

### 3.1. Photophysical Characteristics of Coumarins

**Figure 5:**
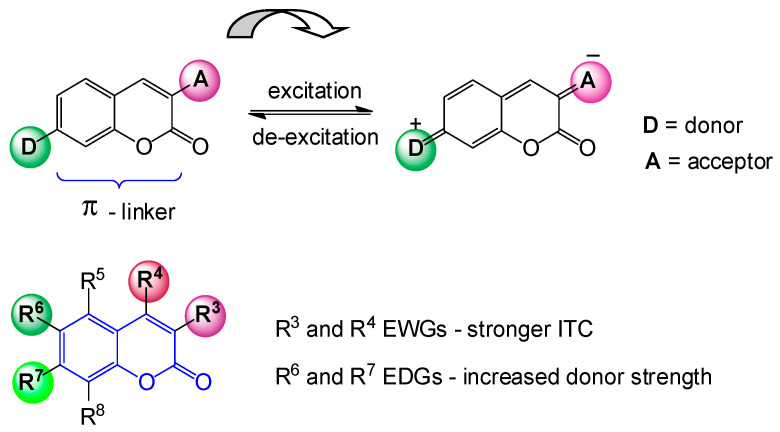
Donor–acceptor architecture of coumarin fluorophores.

Simple coumarin has almost zero fluorescence emission, but appropriate substitution or functionalization of this basic structure can provide derivatives with high fluorescence. The effect depends primarily on the position in which the substituent is introduced and its nature: electron-donating (EDG) or electron-withdrawing (EWG). The presence of a donor–π–acceptor (D–π–A) system gives small molecules like coumarins the ability to fluoresce. Electronic modifications within the D–π–A fragment affect the energy of the edge orbitals and modulate the intensity of the emission. The enhanced push–pull effect, characteristic of dyes acting through intramolecular charge transfer (ICT), leads to a shift in the fluorescence color towards longer wavelengths (Figure 5) [26].

**Figure 6:**
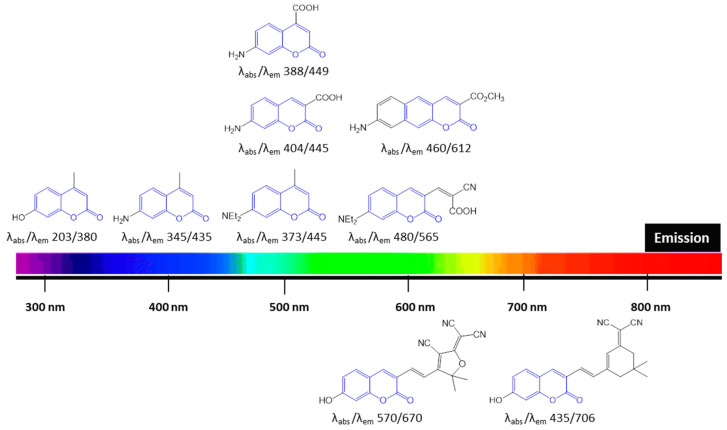
Structures of selected coumarin derivatives and their absorption/emission maxima.

In the coumarin molecule, positions C-4, C-6, and C-7 are of most significance, as they allow effective modulation of the HOMO and LUMO orbital distribution, affecting the color and intensity of the emission. Donor groups (–NH_2_, –OH, –NR_2_) in C-7 increase the electron density of the ring, reduce the HOMO–LUMO gap, and lead to a red shift of the spectrum, as well as improving quantum yield. Acceptor groups (–CF_3_, –COOR, –Cl, –Br) stabilize the LUMO and usually cause a blue shift in emission, with their effect being most pronounced at the C-4 position. Suitable EDGs and EWGs can generate significant red shifts, sometimes at the expense of quantum yield. The polarity of the solvent also modulates the emission properties, influencing both the fluorescence intensity and the Stokes shift value. As a result, even minor modifications of the substituents can precisely tune the absorption and emission, thereby making the coumarin skeleton highly desirable in the development of fluorescent probes. The examples below illustrate how different substituents in the coumarin backbone influence the absorption and emission wavelengths (Figure 6).

### 3.2. Fluorescence Mechanisms of Coumarin Sensors

The photophysical behavior of fluorophores defines how a molecule absorbs and emits light, as well as how it reacts to changes in the chemical medium or the presence of an analyte. Mechanisms such as PeT (photoinduced electron transfer), ICT (intramolecular charge transfer), and ESIPT (excited-state intramolecular proton transfer), which modulate the intensity, color, and efficiency of fluorescence, form the basis of most small-molecule probes. Coumarins are among the fluorophores that are particularly sensitive to these processes due to the presence of a planar benzylidene moiety and the potential for precise substituent modification in the skeleton.

#### 3.2.1. PeT (Photoinduced Electron Transfer)

**Figure 7:**
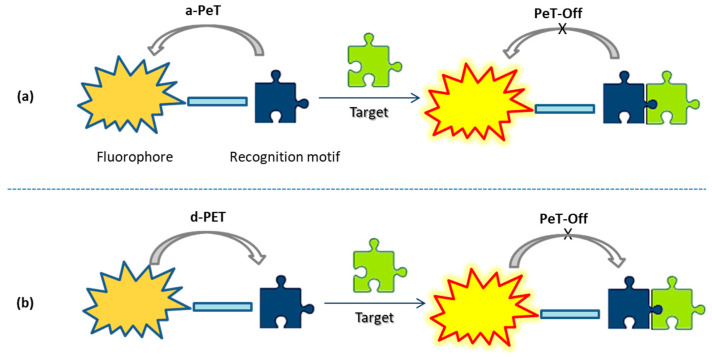
Schematic representation of (**a**) acceptor-excited (a-PeT); (**b**) donor-excited (d-PeT) photoinduced electron transfer mechanisms.

A typical PeT-based fluorescent probe consists of three elements: a fluorophore, a suitable linker, and recognition groups [61]. The PeT mechanism involves photoinduced electron transfer between the donor (D) and acceptor (A) of the probe. In coumarin probes, the donor and acceptor are connected by a π linker, and after excitation of the fluorophore, electron transfer between the donor and acceptor is possible. Based on the direction of electron transfer between the fluorophore and the recognition element, a distinction can be made between acceptor-excited PeT (a-PeT) and donor-excited PeT (d-PeT), Figure 7.

PeT is a particularly important strategy in the design of “off–on” or “on–off” type fluorescent probes. In the initial state, the donor or acceptor group effectively quenches fluorescence through photoinduced electron transfer (PeT-ON). After binding the analyte, the PeT process is inhibited, leading to signal recovery (“off-on”), or conversely, analyte binding can activate PeT and turn off emission (“on-off”). Additionally, the occurrence of PeT is closely related to the chemical properties of the analytes, as they are responsible for changing the HOMO–LUMO energy levels of the recognition group.

PeT-based fluorescent probes are used in the diagnosis and treatment of diseases and in the imaging of intracellular processes such as the detection of reactive oxygen species, gaseous small molecules, biothiols, and biological macromolecules.

**Figure 8:**
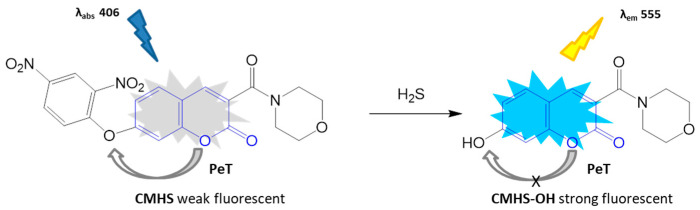
Fluorescence “off–on” switching of the **CMHS** probe induced by H_2_S.

One interesting example of PeT probes is the compound **CMHS** for the selective detection of hydrogen sulfide (H_2_S) [62]. The structure of **CMHS** is based on a coumarin skeleton substituted at the C-7 position with a 2,4-dinitrophenyl group, which is the reaction site with H_2_S. The probe operates on an “off–on” mechanism, activating in the presence of H_2_S, which blocks electron transfer (PeT), leading to strong fluorescence. **CMHS** is characterized by a very fast reaction time, high selectivity, a low detection limit (2.31 × 10^−7^ M), and good membrane permeability (Figure 8). This allows for reliable imaging of both exogenous and endogenous H_2_S in living cells with low cytotoxicity.

**Figure 9:**
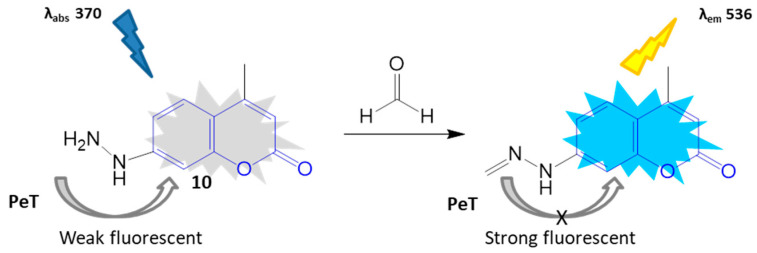
Fluorescence probe **10** for the detection of formaldehyde.

Another example of a coumarin probe operating according to the “turn-on” mechanism is a fluorescent probe for detecting formaldehyde (FA), Figure 9 [63]. In the probe molecule **10**, the hydrazine group quenches the fluorescence of the fluorophore, while the reaction with formaldehyde leads to the formation of a stable imine system, which blocks the quenching process and results in a strong signal enhancement. The probe is characterized by fast reaction time, high selectivity, and low cytotoxicity, and additionally enables imaging of both exogenous and endogenous FA in living cells (HeLa cells).

#### 3.2.2. ICT (Intramolecular Charge Transfer)

**Figure 10:**
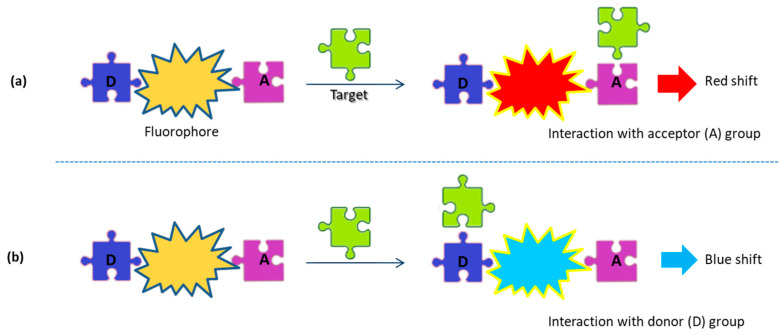
Schematic representation of strong (**a**) and weak (**b**) ICT processes and their effect on fluorescence emission.

Probes based on internal charge transfer (ICT) consist of a fluorophore and the donor group and the acceptor group within a single molecule, which creates a push-pull electron system in the excited state (Figure 10). Under the influence of photoexcitation, charge is transferred from the electron donor to the electron acceptor. The binding of the ICT fluorescent probe recognition group to the analyte affects the push-pull effect of the fluorophore, weakens or strengthens intramolecular charge transfer, and therefore leads to changes in the fluorescence spectrum.

ICT probes are commonly used to detect cations. As a result of the interaction of the electron-donating part with the cation, the electron-donating character of the probe is reduced. This leads to a shift towards the blue end of the absorption spectrum. However, when the cation interacts with the electron-accepting part, a clear shift towards the red of the absorption spectrum is observed due to the greater development of ICT. In addition, changes in fluorescence quantum yield and lifetime are observed.

**Figure 11:**
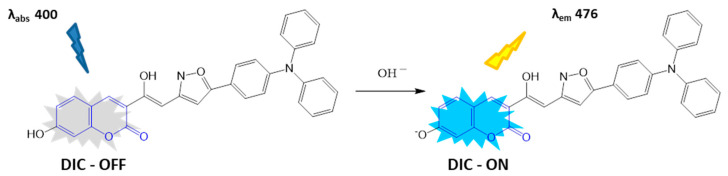
**DIC** probe for real-time detection of pH.

An example of a probe that operates in the ICT mechanism is coumarin **DIC**, 3-(2-(5-(4-(diphenylamino)phenyl)isoxazol-3-yl)-1-hydroxyvinyl)-7-hydroxy-2H-chromen-2-one (Figure 11) [64]. In an alkaline environment, deprotonation of the hydroxyl group leads to enhanced donor properties and increased intramolecular charge transfer efficiency, which further results in enhanced fluorescence. The probe **DIC** could sensitively sense alkaline pH fluctuations in the range of 6.50–9.98 with the pKa value of 8.12 through a remarkable fluorescence turn-on process. The detection method of **DIC** for pH was validated using ^1^H NMR titration and theoretical calculations. **DIC** coumarins were used for pH imaging in zebrafish and for monitoring the freshness of meat and seafood.

#### 3.2.3. ESIPT (Excited-State Intramolecular Proton Transfer)

In excited-state intramolecular proton transfer (ESIPT), photoexcited molecules disperse their energy through tautomerization with proton transfer [65]. Tautomerization often proceeds via keto-enol tautomerism. For coumarin probes, the skeleton should possess an –OH or –NH group that is able to donate a proton, a nearby proton acceptor, and a rigid conjugated system, in which case proton transfer in the excited state will be possible. Under many conditions, ESIPT fluorophores exhibit two emission spectral lines derived from the enol and ketone forms. In addition, ESIPT probes are characterized by a substantial Stokes shift (up to 150–200 nm), long-wavelength emission, and high photostability. Probes of this type are often employed to detect H_2_O, pH, metal ions, viscosity, and biomolecules.

**Figure 12:**
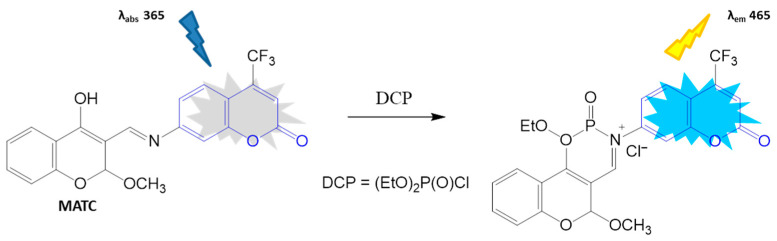
Coumarin **MATC** probe for selective detection of DCP.

An example of an ESIPT probe is the chromone derivative of coumarin **MATC**, which can selectively detect sarin gas mimicking diethylchlorophosphate (DCP) with the detection and quantification limit in the nM range, Figure 12 [66]. In the presence of DCP, the probe displays a bright cyan color fluorescence at a wavelength of 465 nm.

**Figure 13:**
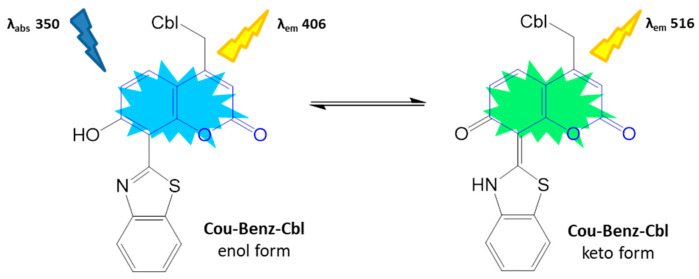
ESIPT-based photophysical behavior of the Cou–Benz-Cbl probe.

An example of a probe based on the ESIPT mechanism and acting simultaneously as a photo-controlled drug delivery system (DDS) is the hybrid coumarin and benzothiazole molecule, **Cou-Benz-Cbl,** Figure 13 [67]. Chlorambucil (Cbl), a cytotoxic anticancer drug, was attached to the probe. The probe exhibits unique photophysical properties, such as good absorption in the range of approximately 350 nm, a large Stokes shift (151 nm), and dual pH-dependent emission (blue-enol form when ESIPT-OFF and green-keto form when ESIPT-ON), which enables cell imaging. Under the influence of UV light, photolytic release of the Cbl drug occurs, leading to apoptosis of MDA-MB-231 cancer cells (breast cancer).

#### 3.2.4. Other Sensing Mechanisms

The mentioned PET, ICT, and ESIPT are the well-studied detection mechanisms employed in the design of coumarin fluorescent probes. In addition to these, it is also worth mentioning FRET (Förster/Fluorescence resonance energy transfer), CHEF (chelation-enhanced fluorescence), and CHEQ (chelation-enhanced quenching), which have emerged in recent years [68,69,70,71].

A technique such as FRET complements the classic strategies of fluorescent probes. The FRET effect consists of non-radiative energy transfer without emitting a photon from one fluorophore (donor) to another (acceptor). To achieve high FRET efficiency, significant overlap between the donor emission spectra and the acceptor absorption bands is typically required. In addition, the donor and acceptor must be in the correct spatial orientation relative to each other and sufficiently close to each other (usually <100 Å). FRET is a tool in the construction of ratiometric probes, distance sensors, and systems that eliminate background autofluorescence. Probes designed in this way are used for macromolecular structure analysis, conformation analysis, and immunoassays [72,73,74].

CHEF is a mechanism in which the binding of a metal ion by a probe rigidifies its structure and inhibits non-emissive energy quenching channels, leading to a significant increase in fluorescence (turn-on). CHEF probes are employed to detect cations with high complexing strength, such as Zn^2+^, Al^3+^, or Mg^2+^ [75,76,77,78]. In CHEQ, on the other hand, metal ion complexation increases the efficiency of quenching processes, which in turn leads to a decrease in the fluorescence signal (turn-off). This mechanism is most commonly observed with heavy metals, e.g., Cu^2+^, Fe^3+^, or Hg^2+^ [79,80,81].

## 4. Coumarin Fluorescent Probes in the Detection of Biological Biomarkers

**Figure 14:**
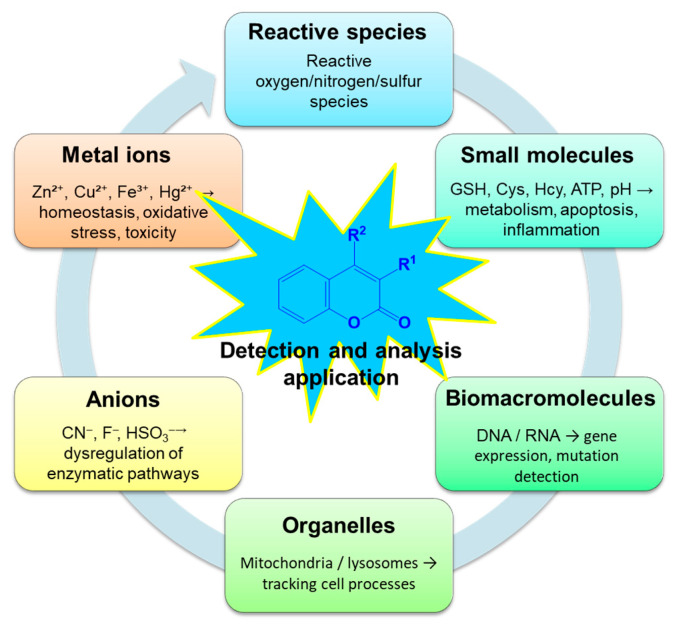
Categories of analytes detected by probes based on coumarin skeleton.

Coumarin fluorescent probes are one of the most important groups of small-molecule analytical tools used in chemistry, biology, and medicine. High quantum yield, high photochemical stability, the possibility of fine structural modification, and compatibility with many sensing mechanisms (PET, ICT, ESIPT, CHEF/CHEQ, FRET) make them very attractive for the design of selective and sensitive sensors. This chapter presents a classification of coumarin probes according to the detected analytes, including inorganic compounds (metal ions, anions), reactive oxygen and nitrogen species, cellular biomarkers (H_2_S, formaldehyde, biothiols), and organelle (Figure 14). We discuss the most important strategies for probe construction and provide examples of the latest solutions reported in the literature since 2023.

### 4.1. Coumarin-Based Probes for Biologically Relevant Small Molecules

Small endogenous molecules such as thiol amino acids (GSH, Cys, Hcy), ATP and ADP, formaldehyde, and pH change signals play a key role in regulating metabolism, oxidative stress, apoptosis, and cancer processes. Precise monitoring of their levels in vitro and in vivo may be of significant diagnostic importance for many diseases.

#### 4.1.1. Biothiol Detection

Cysteine (Cys), homocysteine (Hcy), and glutathione (GSH) are amino acids with strong reducing and nucleophilic properties [82,83,84]. They protect cells and tissues from endogenous reactive oxygen species (ROS) and free radicals [85]. These biothiols are involved in the regulation of cell signaling, cell life cycle, apoptosis, protein biosynthesis, and immune homeostasis.

Cysteine is the main source of sulfur in human metabolism and is essential for maintaining the tertiary and quaternary structure of proteins. It is also a precursor of glutathione and supports the maintenance of cellular redox homeostasis [86,87,88,89]. The physiological concentration of Cys in humans is 30–200 μmol/L, and both excess and deficiency of Cys can result in serious disorders. Enhanced Cys levels are associated with rheumatoid arthritis, Parkinson’s disease, and Alzheimer’s disease [90,91], while deficiency causes developmental delays, skin, and liver damage [92,93].

Homocysteine (Hcy) is a key biomarker of metabolic disorders. In healthy adults, the physiological concentration is 9–13 μmol/L, while values above 15 μmol/L are classified as hyperhomocysteinemia, a disorder strongly associated with cardiovascular disease (heart attack, stroke), neurodegeneration, and DNA methylation disorders. Hcy can lead to atherosclerosis by damaging the inner lining of blood vessels. High homocysteine levels are also associated with Alzheimer’s disease, dementia, and diminished cognitive function [94,95,96,97,98].

Glutathione (GSH) is the most abundant non-protein amino acid, ranging in concentration from 1 to 10 mmol/L, and playing a fundamental role in controlling oxidative stress, detoxification processes, and apoptosis regulation. Glutathione is a key antioxidant, and its deficiency can lead to increased damage induced by free radicals. GSH level disorders are observed in pathological conditions such as AIDS, cancer, liver disease, cystic fibrosis, anemia, chronic lung disease, and diabetes [99,100,101,102].

To date, many fluorescent probes have been developed for the detection of compounds containing thiol groups [103,104,105,106,107,108,109,110,111,112,113,114,115,116]. When designing such probes, the reactivity of the –SH group, which is a strong nucleophile, is important to consider. The most commonly employed mechanisms in probe construction involve:

(i) Michael addition to α,β-unsaturated carbonyl systems, in which the thiol reacts with the conjugated C=C bond, leading to changes in the π conjugation and a noticeable change in emission; (ii) nucleophilic substitution in probes bearing a good leaving group (halogen, ether, thioether), which allows the unblocking of a masked donor group on the coumarin skeleton and the activation of the probe (turn-on); (iii) cleavage of sulfonamide or sulfonate ester, nucleophilic attack of –SH leads to the release of a free phenolic coumarin group or its active fluorophore; (iv) disulfide–thiol exchange reaction, which occurs rapidly and selectively under biological conditions; and (v) other reactions utilizing the unique reactivity of the –SH group.

**Scheme 2:**
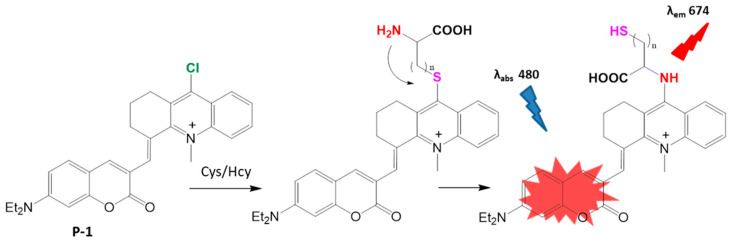
Mechanism of action the coumarin hybrid tetrahydro-acridine salt probe.

To detect Cys/Hcy, a compound based on the coumarin hybrid tetrahydro-acridine salt was developed [117]. Probe **P-1** demonstrated excellent sensitivity and quick response times and more importantly, shows a clear turn-on response in the near-infrared region, with an emission maximum around 674 nm and a notably large Stokes shift of approximately 200 nm upon reaction with Cys or Hcy (Scheme 2). In addition, the system functions effectively in biological settings, enabling selective visualization of Cys/Hcy within mitochondrial compartments of living cells as well as in vivo.

**Scheme 3:**
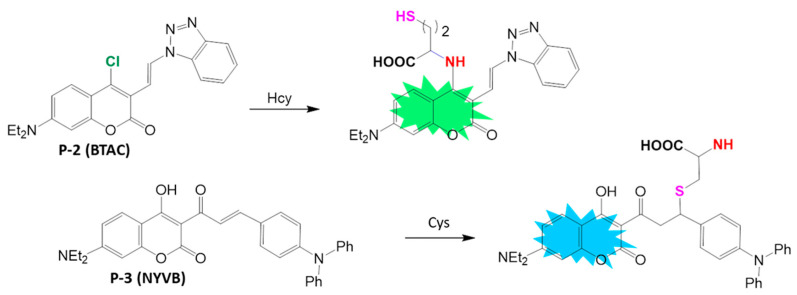
Coumarin probe for detection of Cy and Hcy.

In 2023, Li et al. [118] reported a benzotriazole-coumarin hybrid **BTAC** (**P-2**) that demonstrated high selectivity toward Hcy (Scheme 3). The probe reacted with homocysteine in a nucleophilic halogen substitution mechanism combined with a rearrangement reaction, leading to a significant enhancement of fluorescence. The presence of the benzotriazole moiety significantly increases its response rate to Hcy. Under optimized conditions, the fluorescence intensity of the probe was linearly correlated with Hcy concentration in the range of 2.0 × 10^−7^–7.0 × 10^−6^ mol/L, with a detection limit of 1.55 × 10^−8^ mol/L. Furthermore, this coumarin was successfully used for imaging homocysteine in living cells.

The **NYVB** probe has been successfully employed for the determination of Cys in real food and serum samples (Scheme 3) [119]. After binding with the analyte, the fluorescence intensity of the probe increased 55.7-fold. **NYVB** has been successfully used to detect endogenous and exogenous Cys in SKOV3 cells (human ovarian cancer cells) and to track Cys detection in redox imbalances induced by H_2_O_2_, Hg^2+^, or Cu^2+^.

**Scheme 4:**
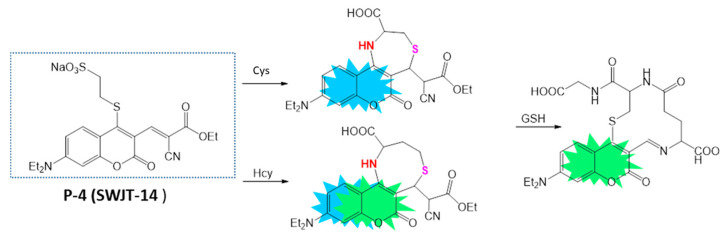
Detection in multiple excitation channels with probe **SWJT-14**.

The Wang group synthesized **SWJT-14** [120], which proved to be a versatile tool for the differential detection of three biothiols (Scheme 4). Its action is based on a chemodosimetry mechanism, in which the reaction with the thiol group leads to the formation of different structural products. These differences generate characteristic absorption and fluorescence signatures, enabling selective detection of analytes in aqueous solutions and in living cells. Depending on the type of biothiol, the reaction products of the **SWJT-14** probe display different fluorescence profiles. When excited at 380 nm, cysteine exhibits intense emission at 470 nm, while homocysteine and glutathione show weak fluorescence at 467 nm and 481 nm, respectively. In turn, at 460 nm excitation, a characteristic maximum of 550 nm occurs selectively for homocysteine, while 490 nm excitation leads to 553 nm emission, typical for the reaction product with glutathione.

The mechanism of operation of the probe involves an initial nucleophilic attack of the –SH group on the conjugated electron system of **SWJT-14** and displacement of the 2-mercapto sulfonate group, leading to the formation of the thiol adduct. This is followed by rapid intramolecular rearrangement to a Schiff base, which in the case of Cys and Hcy proceeds further through Michael reaction and cyclization, leading to polycyclic, strongly conjugated products. In the case of GSH, due to high steric hindrance, cyclization does not occur. **SWJT-14** exhibits very low intrinsic fluorescence (Φ = 0.32%, 0.26%, and 0.06% depending on excitation wavelength); however, upon reaction with biothiols a pronounced turn-on response is observed, with quantum yields increasing to Φ = 2.8% for cysteine, Φ = 2.3% for homocysteine, and Φ = 1.67% for glutathione in PBS buffer (pH 7.4). Due to its high sensitivity, fast reaction times (especially towards Cys), and the possibility of detection in multiple excitation channels, **SWJT-14** is one of the most advanced and practical coumarin probes developed in recent times.

**Scheme 5:**
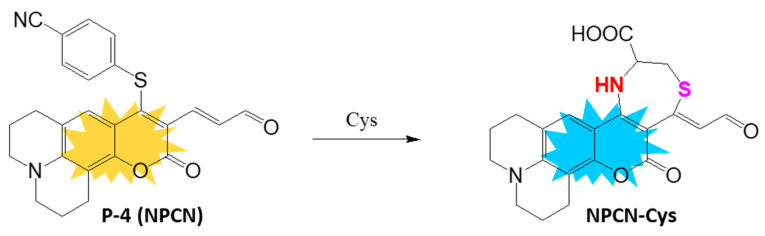
Ratiometric fluorescent probe, **P-4** (**NPCN)**.

The ratiometric fluorescent probe, **P-4** (**NPCN)**, was developed for the detection of cysteine [121]. Ratiometric probes are an advanced group of fluorescent analytical tools in which analyte detection is based on a change in the ratio of the intensity of two independent optical signals. In contrast to classic turn-on/turn-off probes, ratiometric systems minimize errors related to local fluorophore concentration, lighting heterogeneity, varying sample thickness, and instrumental fluctuations. Consequently, the data generated is more reliable and reproducible, which is important in biological imaging. Due to the presence of two reactive sites, the reaction of the probe **NPCN** with Cys results in a change in the intensity ratio of the two fluorescent emissions, with a clear shift in the emission maximum from 590 nm to 472 nm (Scheme 5). High selectivity towards Cys and a low detection limit (0.18 μM) enabled the authors to image Cys both in cells and in live zebrafish.

**Scheme 6:**
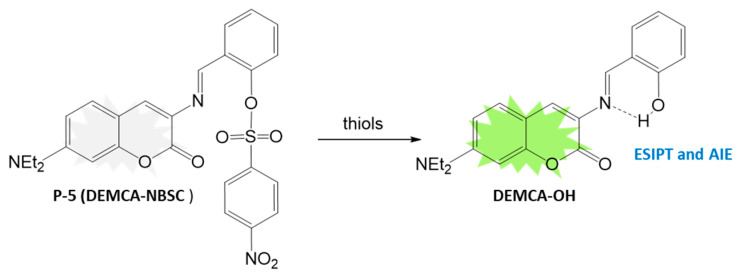
Activation of **DEMCA-OH**, synergistic effect of ESIPT and AIE.

Another probe for detecting thiols, **P-5** (**DEMCA-NBSC)**, was constructed from a derivative of 7-dimethylamino coumarin, which acts as an electron donor (D), and a *p*-nitrobenzenesulfonyl (NBSC) group, which is a strong acceptor (A) [122]. The presence of the NBSC group effectively inhibits two key photophysical mechanisms of the fluorophore—ICT and ESIPT—and additionally suppresses the aggregation-induced emission (AIE) phenomenon, as a result of which the probe does not emit light (Scheme 6). Coumarin has a D–A character, which promotes photoinduced electron transfer (PET). In the presence of thiol, selective and fast (≤3 min) hydrolysis of the sulfonate bond and cleavage of the NBSC group proceed. The free **DEMCA-OH** probe achieves ESIPT capability, and intramolecular and intermolecular hydrogen bonds are formed, activating the process of aggregation-induced emission. Under the influence of UV-365 nm, strong fluorescence is observed in the yellow-green range. The NBSC probe is characterized by high selectivity, low detection limits (Cys 0.236 μM; GSH 0.223 μM; Hcy 0.365 μM), and an appropriate Stokes shift (~110 nm), which enables biological imaging and minimizes autofluorescence. It has been used for biothiol detection in vitro and in vivo imaging due to its low cytotoxicity.

**Scheme 7:**
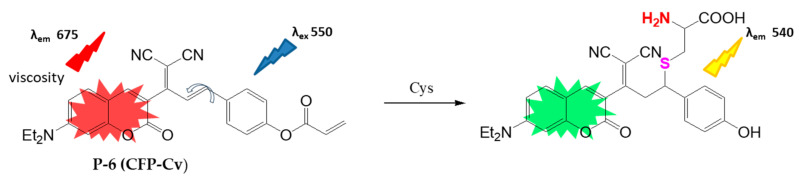
Illustration of the fluorescence turn-on/shift response of the coumarin **P-6**.

**CFP-Cv** (**P-6**) is a dual-channel fluorescent probe designed for monitoring both cysteine levels and viscosity changes in cells during erastin-induced ferroptosis [123]. Ferroptosis is iron-dependent apoptosis involving the accumulation of lipid peroxides and oxidative stress [124]. The C=C bond beyond the coumarin skeleton is sensitive to viscosity changes (by inhibiting molecular rotation), while the acrylic group at C-7 of the skeleton reacts selectively with Cys. Under low viscosity, or in the absence of analyte, fluorescence emission is reduced (ICT-suppressed). According to the authors, in the presence of Cys, a Michael reaction occurs and the acrylic residue is removed, resulting in a marked increase in fluorescence emission at 540 nm. Independently, an increase in the viscosity of the environment limits intramolecular rotation, activating emission in the red range (λ_em_ = 675 nm, λ_ex_ = 500 nm), (Scheme 7). The probe demonstrates high selectivity towards Cys and low cytotoxicity and enables visualization of biochemical and biophysical changes in A549 cells subjected to ferroptotic stress.

**Scheme 8:**
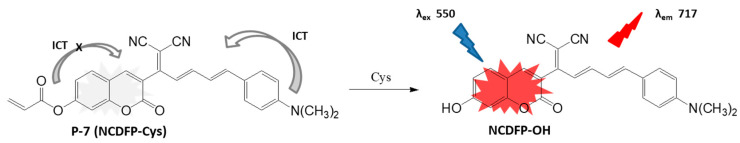
Proposed detection mechanism of **NCDFP-Cys** for cysteine.

In 2024 Shao’s group [125] reported the **NCDFP-Cys** probe (**P-7**), which was synthesized from 3-acetyl-7-hydroxycoumarin. To generate emission in the near-infrared range, a malonitrile group (electron acceptor) and 4-(dimethylamino)cinnamaldehyde, a strong electron donor, were introduced into the coumarin skeleton (Scheme 8). In the **NCDFP-Cys** probe, the ICT process is inhibited by the acrylate group at the C-7 position. The presence of cysteine leads to the detachment of the acrylic moiety, and the 7-OH group (**NCDF-OH** with a D–π–A–π–D structure) is unlocked, and ICT activity returns, which manifests as strong emission in the near-infrared range (NIR, λ_em_ = 717 nm, λ_ex_ = 550 nm), Scheme 7. The authors reported a low quantum yield for NCDFP-Cys (Φ = 0.022 in DMSO–PBS, 8:2, pH 7.4), which results from ICT quenching by the acrylate moiety, whereas the reaction product NCDF-OH exhibits a markedly increased fluorescence efficiency (Φ = 0.134 under the same conditions). In addition, the probe showed a low detection limit (LOD = 0.14 µM), fast response (emission within 5 min), and large Stokes shift (167 nm). The probe was used to visualize endogenous and exogenous cysteine levels in living cancer cells (SW1990 line) and to monitor changes in Cys concentrations in response to two different ferroptosis inducers—erastin (which lowers Cys levels) and RSL3 (which acts independently of Cys levels).

**Scheme 9:**
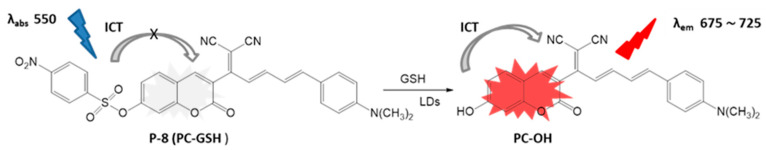
Dual-locked probe **CP-GSH** for tissue imaging.

Another probe presented by Shao’s group is a dual-locked coumarin derivative **P-8** (**PC-GSH**) that requires two independent stimuli to generate a fluorescent signal (Scheme 9) [126]. This **PC-GSH** probe is activated by high concentrations of glutathione (GSH) and high viscosity microenvironments, lipid droplets (LDs). The **PC-GSH** design consists of a π-expanded coumarin (**PC-OH**) as the fluorophore and a DNS moiety (2,4-dinitrobenzenesulfonate) as the recognition group for GSH. The simultaneous presence of GSH and LDs produces a strong signal in the 675–725 nm range. In vitro and in vivo imaging studies confirmed that **PC-GSH** has low cytotoxicity, excellent photostability, and a distinct ability to localize lipid droplets. In addition, the probe effectively distinguished cancer cells from normal cells based on differences in GSH and LDs levels. With emission in the NIR range, **PC-GSH** is suitable for deep tissue imaging.

**Figure 15:**
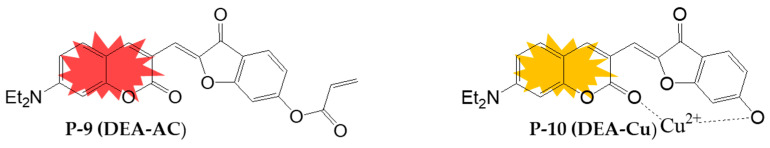
Fluorescent probes, **DEA-AC** (**P-9**) and **DEA-OH-Cu^2+^** (**P-10**).

Recently, Cao et al. designed and synthesized two fluorescent probes, named **DEA-AC (P-9)** and **DEA-OH-Cu^2+^** (**P-10**) both bearing a coumarin-auron skeleton for Cys detection, Figure 15 [127]. The **DEA-OH-Cu^2+^** probe works on the basis of a classic off–on assay: the Cu^2+^ ion strongly quenches the fluorescence of the complex, while after reaction with Cys, nucleophilic substitution occurs and an intensely fluorescent form of DEA-OH is released (λ_em_ 596 nm). In turn, the coumarin DEA-AC, the reactivity of the acrylate group, is used; in the presence of Cys, Michael addition and intramolecular cyclization take place, leading to the formation of DEA-OH and a strong fluorescence signal shifted from 659 to 616 nm. DEA-AC is characterized by a significantly faster response (3.5 min), higher sensitivity, and enhanced selectivity towards Cys compared to DEA-OH-Cu^2+^. The fluorescence detection limits (LOD) for **DEA-AC** and DEA-OH-Cu^2+^ are 1.65 μM and 7.25 μM, respectively. The fluorescence quantum yield of DEA-OH–Cu^2+^ increased from Φ = 0.049 to Φ = 0.31 upon reaction with cysteine, while the acrylate-masked probe DEA-AC exhibited a quantum yield of Φ = 0.076 that rose to Φ = 0.29 after Cys-triggered acrylate hydrolysis (DMSO/HEPES, 1:1, pH 7.4). Both probes were used in bioimaging studies in living HeLa cells for the detection of cysteine after evaluating their cytotoxicity and cellular permeability.

**Figure 16:**
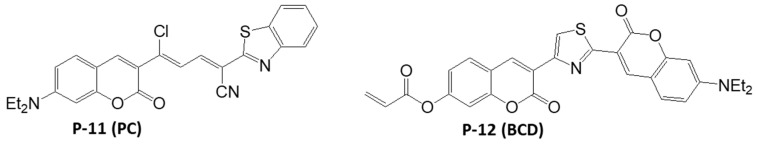
Fluorescent probes, **PC** and **BCD**.

Figure 16 illustrates fluorescent probes designed for selective detection of biological thiols (Cys, Hcy, GSH) or hydrazine, whose design was based on a donor–π–acceptor strategy, enhancing the push–pull effect and fluorescence emission in the visible range. The **PC** (**P-11**) probe is a hybrid of coumarin and thiazole bearing an acryloyl ester group as the triggering and responding entity for the identification of cysteine [128]. After the Michael reaction and cyclization with Cys, the acryloyl ester is cleaved, releasing a strongly emitting **PC–OH** fluorophore (λ_em_ = 508 nm, λ_ex_ = 375 nm). The probe is characterized by a large Stokes shift (133 nm). **PC** was employed for the quantitative determination of Cys in real food samples (milk, powdered milk, cookies, apples, and onions), in A549 cells (adenocarcinoma of the lung), as well as in vivo in zebrafish larvae, where the signal clearly increases after the addition of Cys and disappears after thiol blocking.

In turn **BCD** (**P-12**) is a multi-site fluorescent probe developed for the simultaneous and selective detection of three biothiols and hydrazine (N_2_H_4_) in aqueous-organic solutions [129]. The probe design employs a coumarin skeleton and benzothiazole. The four reactive sites in BCD allow the analytes to be distinguished based on different nucleophilic reaction mechanisms: nucleophilic aromatic substitution (SNAr), Michael addition, and addition to the cyano group. After reacting with the analyte, the probe exhibits marked, characteristic changes in fluorescence: Cys gives a 760-fold increase in emission at 502 nm (λ_ex_ = 463 nm), Hcy—an 8-fold increase at 479 nm (λ_ex_ = 396 nm), and GSH—a slight increase at 476 nm (λ_ex_ = 365 nm), while hydrazine generates a strong emission at 458 nm (λ_ex_ = 407 nm) with an intensity increased by ~637 times.

**Figure 17:**
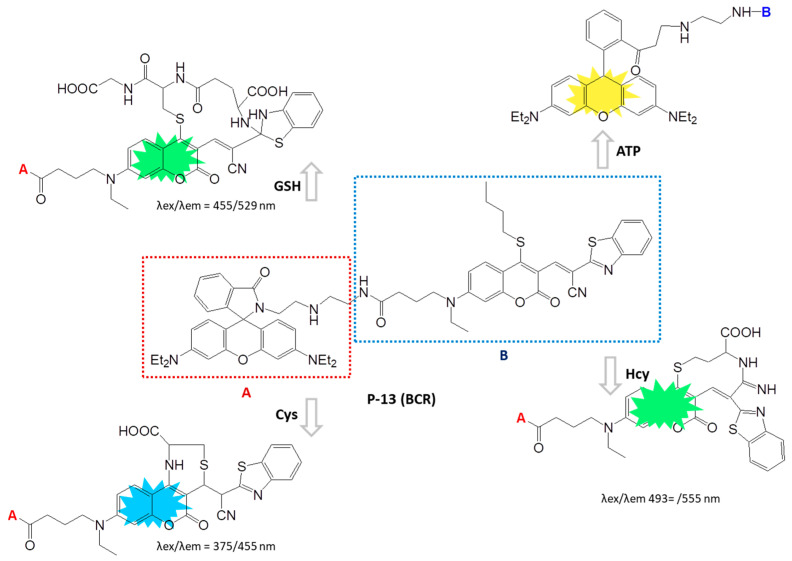
The action of multifunctional probe **BCR** (**P-13**).

A very attractive and complex probe is the multifunctional molecule **BCR** (**P-13**) [130]. The compound has a coumarin skeleton which is selective towards Cys, Hcy, and GSH, and a rhodamine skeleton which reacts exclusively with ATP. Both fragments are connected by a piperazine linker, which ensures high selectivity and no interference between the two skeletons. Thanks to this design, the **BRC** probe enables the simultaneous detection of four biomarkers with completely separate emission bands (455, 529, 555, and 587 nm), Figure 17. Despite its high molecular weight, **BCR** is characterized by very good biocompatibility and has been successfully used in models of pentylenetetrazole (PTZ)-induced epilepsy in SH-SY5Y cells (human cell line of nervous system cancer) and zebrafish larvae. PTZ blocks the GABA-A receptor, causing excessive neuronal activity and oxidative stress, which leads to characteristic changes in biothiol and ATP levels. Through multi-channel fluorescence, the probe enabled simultaneous monitoring of the decrease in GSH, Cys, and ATP and the increase in Hcy, thus demonstrating the scale of damage in both models.

**Figure 18:**
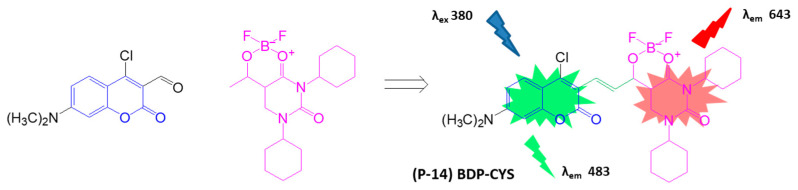
Synthetic Approaches for **BDP-CYS** probe.

The classic ratiomeric near-infrared (NIR) probe **BDP-CYS** (**P-14**) is designed to detect biothiols (Cys, Hcy, GSH) in living cells and animal models [131]. The structure of **BDP-CYS** includes: a chlorinated coumarin derivative conjugated with hemicyanine, which is reactive with thiols, and dioxaborine–barbiturate (BODIPY derivatives), which enables intense emission in the red range and increased brightness (Figure 18). In addition, large cyclohexyl bases have been introduced into the probe structure to prevent aggregation. The mechanism of action of the probe is based on SNAr on the chlorine atom in the coumarin skeleton, initiated by the –SH groups of biothiols. The reaction causes the π coupling between the coumarin unit and BODIPY to break, resulting in a decrease in BODIPY (red) emission and a simultaneous increase in coumarin (green) emission. This allows for precise ratiometric reading.

The **BDP-CYS** probe has been successfully employed for highly sensitive, ratiometric detection of biothiol in cell models, cells, zebrafish larvae, and mice. It allows not only the detection of endogenous thiols, but also monitoring of liver damage and evaluating the effectiveness of therapy with *N*-acetylcysteine in real time. Due to its NIR emission (643 nm), low toxicity, and strong ratiometric signal, **BDP-CYS** has unique application potential in functional imaging of mammalian organs.

#### 4.1.2. Formaldehyde Detection

Formaldehyde (FA) is a reactive organic compound that is considered a potential carcinogen and is associated with many chronic diseases, including cardiovascular disease, diabetes, cancer, and neurodegenerative diseases [132,133]. Its concentration varies both in the environment and in the human bodys with a biological half-life of approximately 1 h. FA readily reacts with functional groups such as –OH, –SH, and –C=O, as well as with molecules present in the biological environment, including water, ethanol, and amino acids. This high reactivity leads to the existence of formaldehyde in many chemical forms: monomer, diol (methanediol), polymers (paraformaldehyde), and trioxane.

FA detection requires specific reactive groups such as: homoallylamine (aza-Cope reaction), primary amines (condensation to Schiff base), hydrazines (PET block after Schiff base formation), β-diketones/β-ketoesters (Hantzsch reaction). Typical fluorophores used for FA detection are: furazans, BODIPYs, naphthalimides, imidazoles, benzimidazoles [134,135,136,137,138,139,140]. Probes for formaldehyde detection based on a coumarin skeleton are uncommon [139,141,142,143,144], which indicates a significant research gap and potential innovative advantage.

**Scheme 10:**
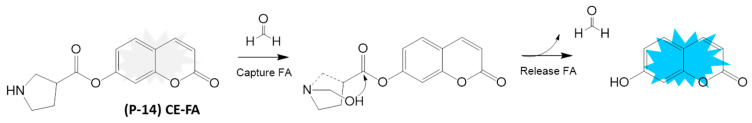
**CE-FA** (**P-15**) probe designed for detection of FA.

In 2024, Zhu et al. designed a **CE-FA** (**P-15**) probe based on a coumarin fluorophore conjugated with pyrrolidine-3-formate, which acts as the FA recognition site (Scheme 10) [145]. After formaldehyde binds to the pyrrolidine group, alcoholization of the ester group occurs, which simultaneously leads to the release of free fluorophore (increase in fluorescence signal) and the reformation of the FA molecule. This mechanism ensures that detection does not affect the FA equilibrium in the biological system, which distinguishes **CE-FA** from classical analyte-consuming probes.

The **CE-FA** probe presents high selectivity and sensitivity, and its emission after reaction with FA intensifies significantly at λ_em_ = 454 nm (blue/greenish fluorescence). The detection limit is 2.6 μM, which is sufficient for the determination of FA in environmental and food samples. **CE-FA** has also been successfully used for imaging endogenous and exogenous formaldehyde in live zebrafish larvae.

**Scheme 11:**
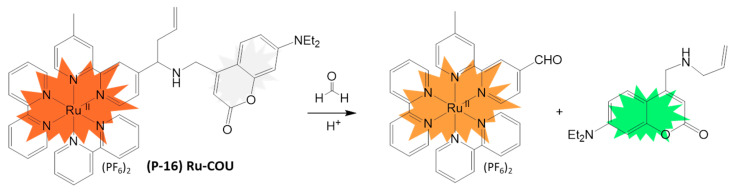
The response mechanism of **Ru-COU** probe.

Recently, a ratiometric **Ru-COU** (**P-16**) probe was also presented, structured from coumarin and a tris(bipyridyl)ruthenium(II) complex connected by an allylamine linker reactive with formaldehyde. The presence of the Ru(II) complex ensured red emission of the probe, while the presence of coumarin ensured fluorescence activated by a reaction with the analyte. The response mechanism is based on the condensation reaction of FA with the amino group, followed by the aza-Cope reaction [146,147], which restores the emission of the coumarin moiety while weakening the luminescence of the Ru(II) complex (Scheme 11). This results in a strong ratiometric signal with a large emission shift >100 nm and an ~400-fold increase in intensity after the addition of the analyte.

The **Ru-COU** probe works exclusively in an acidic environment (pH 3–6), such as that found in lysosomes, and is highly selective towards FA. The detection limit is 17.7 μM, and the full reaction time is approximately 120 min. The authors conducted in-cellulo studies (on a human nerve cell line, SH-SY5Y) and on mouse tissues, which showed the probe’s potential for visualizing endogenous FA in lysosomes. In addition, high levels of FA were recorded in the brains of mice with Alzheimer’s disease (APP/PS1), characteristic of neurodegeneration.

#### 4.1.3. NADH and NADPH Detection

NADH is a key regulator of redox reactions in living cells and one of the coenzymes of energy metabolism. It plays a role in both cytoplasmic glycolysis and the mitochondrial respiratory chain, where it participates in the transfer of electrons necessary for ATP synthesis. The normal NAD^+^/NADH ratio is strictly controlled because it can lead to the deregulation of redox homeostasis and numerous metabolic disorders. Enhanced NADH levels are typical for cancer cells, which use increased anaerobic glycolysis (Warburg effect) and altered oxidative phosphorylation to supply their increased energy demand [148,149]. For this reason, NADH is considered an important biomarker of metabolic processes, oxidative stress, cancer progression, and other diseases associated with energy pathway disorders.

NADPH, on the other hand, is responsible for preserving redox balance and ensuring that cells respond properly to oxidative stress. Low NADPH levels result in increased ROS production and cellular damage. NADPH deficiency also affects glucose metabolism and impairs insulin secretion, which is one of the mechanisms involved in the development of type 2 diabetes [150,151,152,153]. NADPH is therefore a biomarker for metabolic processes and diseases associated with energy dysfunction and oxidative stress.

Probes for NADH/NADPH detection should contain units with high electron-accepting ability (quinone, resazurin, pyridine or quinoline cations) and allowing rapid reduction by NADH/NADPH to generate a turn-on signal and additionally exhibit selectivity over other reducers: GSH, Cys, AA, FADH_2_. Fluorophores based on hemicyanines, rhodamine, resazurin, and benzophenoxazine, dominate, providing emission in the red or NIR range and good cellular permeability, making them suitable for in vitro and in vivo imaging [154].

**Scheme 12:**
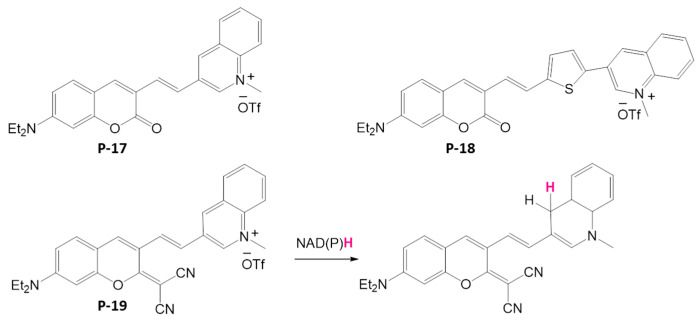
The coumarin probes for detection of NAD(P)H.

Recently, Liu et al. presented three probes based on coumarin conjugated with a 3-quinoline acceptor, which together form a D–π–A system with tunable acceptor strength [155]. Probes **P-17** and **P-18** are based on the coumarin framework, in which the coumarin core is connected by a vinyl bond (**P-17**) or a thiophene bridge (**P-18**) to a cationic quinolinium unit, while probe **P-19** contains dicyanomethylidene group in place of the lactone carbonyl, which increases the electron-accepting ability of the system and enhances the reaction with NAD(P)H. All three probes present strong emission quenching in the initial state due to the deactivation of ICT charge transfer. After hydrogen transfer from NAD(P)H, the quinolinium group is reduced to 1-methyl-1,4-dihydroquinoline, which switches the system to D–π–A–π–D, enhances ICT efficiency, and generates a turn-on signal (Scheme 12).

The maximum emissions of the probes are in the range of 535–583 nm, depending on the structure, with probe **P-19** reporting the most favorable properties and fast reaction kinetics. The probes were used to monitor NAD(P)H levels in living cells, including HeLa cells and fruit fly larvae.

#### 4.1.4. H_2_S Detection

H_2_S is the smallest bioactive thiol that acts as a gaseous transmitter (similar to NO and CO) [156]. In low concentrations, it regulates numerous physiological processes (neurotransmission and anti-inflammatory response), while its excess results in toxic inhibition of cellular respiration by binding to the iron centers of mitochondrial enzymes. Due to its wide range of biological concentrations (nM–µM) and its important role in physiology and pathology, the development of sensitive and selective fluorescent probes for H_2_S detection has become an important area of research [157,158].

Fluorescent probes for H_2_S detection exploit the high nucleophilicity and reducing properties of HS^−^. The presence of reactive groups such as azides, nitro- and hydroxylamines, DNP/DNBS/NBD groups, electrophilic chlorides, and cyanides, which undergo reduction, nucleophilic substitution, or thiolysis under the influence of H_2_S, is desirable.

High-sensitivity fluorophores are used in H_2_S detection probes: derivatives of quinoline, coumarin, rhodamine, chromene, BODIPY, carbazole, and hemicyanine emitting in the red and NIR [159,160,161,162,163,164,165]. The probes are dominated by classic photophysical pathways: ICT, PET, FRET, and ESIPT, which determine whether the signal is “turn-on,” “turn-off,” or ratiometric. These mechanisms are activated in response to a change in the electronic nature of the molecule after reaction with H_2_S, e.g., as a result of the conversion of an azide group to an amine group, the breaking of a π bond, or the formation of a ring after nucleophilic addition.

**Figure 19:**
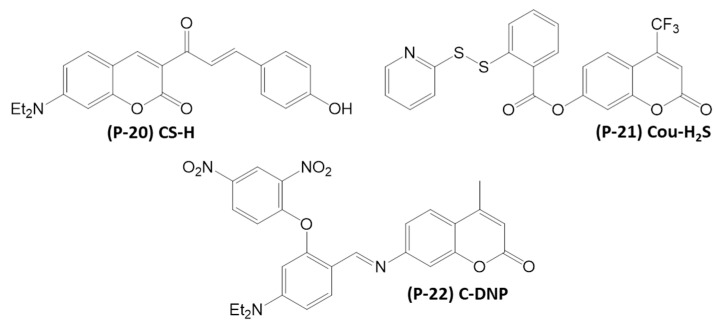
Coumarin derivatives for detection of H_2_S.

Figure 19 illustrates fluorophores **P-20-22** for thiol detection based on a coumarin skeleton. The **C-HS** (**P-20**) probe is a hybrid of coumarin and chalcone, whose fluorescence is completely quenched by the 2,4-dinitrobenzenesulfonyl (DNBS) group [166]. DNBS recognizes the analyte and effectively blocks charge transfer (PET). After interaction with H_2_S, the DNBS group is cleaved and the fluorophore is activated (λ_em_ = 575 nm, λ_ex_ = 470 nm). The fluorescence quantum yield increases from 0.01 (probe alone) to 0.20 after activation with H_2_S (60 μM; PBS/DMSO 8:2, pH 7.4). The probe was used for the detection of endogenous H_2_S in living HeLa cells.

The **Cou-H_2_S** (**P-21**) probe, on the other hand, was designed based on 4-trifluoromethyl coumarin, and 2-pyridyl disulfide was used as the H_2_S recognition group [167]. In the native state, the fluorescence of the fluorophore is inhibited, with the disulfide group masking the emission. After reacting with the analyte, the **Cou-H_2_S** probe emits intense green fluorescence at 498 nm (λ_ex_ 405 nm), with the signal increasing more than 300-fold, allowing detection with a limit of 25 nM. Under biological conditions, it allows imaging of both exogenous and endogenous H_2_S in HeLa cells and monitoring of changes in H_2_S levels in RAW264.7 inflammatory cells (mouse macrophages) stimulated with lipopolysaccharide causing inflammation.

The synthesis of the **C-DNP** (**P-22**) probe uses a 2,4-dinitrophenyl (DNP) group, which acts as a strong electron acceptor and quencher [168]. The presence of DNP blocks the ICT process and causes the probe to exhibit weak fluorescence in its initial state. H_2_S reacts with DNP-C–O, breaking the ether bond and unblocking effective ICT. Consistently, the fluorescence quantum yield increases from 0.21 for the intact probe to 0.68 after reaction with H_2_S, corroborating the efficient fluorescence activation upon DNP cleavage. The probe has been successfully used to image H_2_S in intestinal epithelial cells (NCM460) and to distinguish breast cancer cells from normal cells.

**Scheme 13:**
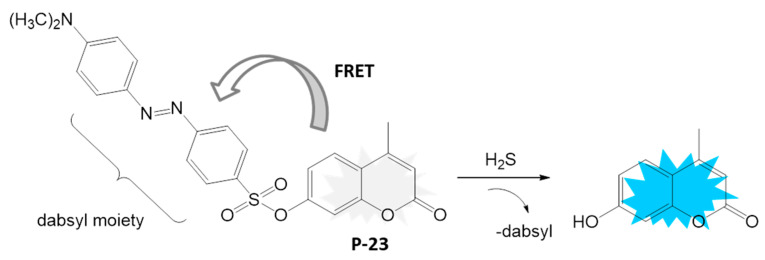
Reaction mechanism of the dabsyl–coumarin off-on.

The Thongyoo group developed a classic *on-off* H_2_S detection probe **P-23** composed of 7-hydroxy-4-methylcoumarin acting as a fluorophore and a dabsyl group acting as a strong dark quencher [169]. Both elements were connected by an *O*-sulfonyl bond, creating a FRET system in which the fluorescence of coumarin is completely quenched (fluorescence-off), Scheme 13. The mechanism of H_2_S detection was based on the thiolysis of the sulfonate bond and the release of coumarin (fluorescence-on). The **P-23** probe is characterized by good stability, lack of cytotoxicity, and cell permeability, which allowed the authors to employ it for imaging H_2_S levels in living HeLa cells.

**Figure 20:**
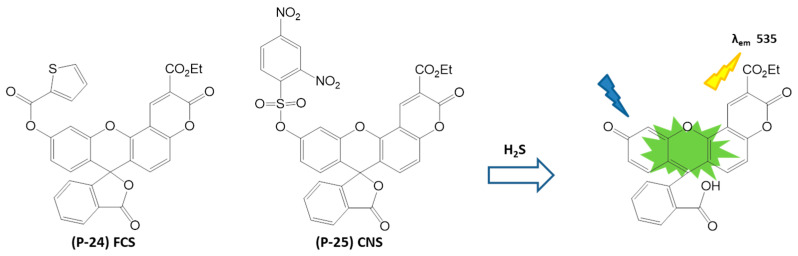
**FCS** (**P-24**) and **CNS** (**P-25**) probes.

Two dual, newly designed **FCS** (**P-24**) and **CNS** (**P-25**) probes are shown in Figure 20 [170]. These molecules were composed of a coumarin and fluorescein skeleton to extend the emission range and improve photophysical properties. The probes operate on a turn-on mechanism, activated by the nucleophilic thiolysis of masking groups in the presence of the analyte.

Of particular interest is the **FCS** probe, which reacts with H_2_S in <1 s, making it a probe with exceptionally fast H_2_S response kinetics. In response to H_2_S, **FCS** emission shifts to 535 nm, characterized by a large Stokes shift (~85 nm) and a more intense signal compared to probes based on fluorescein alone. **FCS** and **CNS** display high stability, are simple to synthesize, and are selective for H_2_S. Both molecules have been used to monitor endogenous and exogenous H_2_S in HeLa cells, as well as for imaging in live zebrafish larvae. The **FCS** probe has also been used in a *Drosophila* model of Parkinson’s disease, where it accurately reflected changes in H_2_S levels associated with the activity of the enzyme cystathionine *β*-synthase (CBS).

**Scheme 14:**
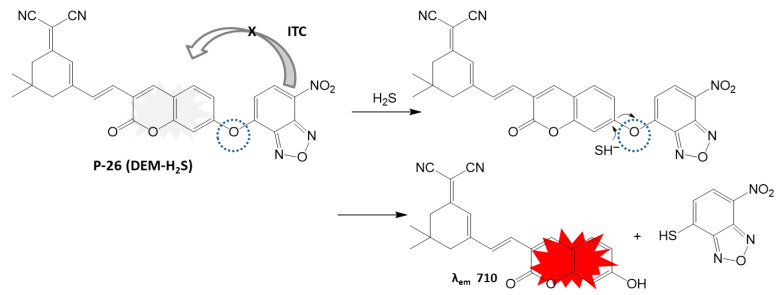
Mechanism of action probe **DEM-H_2_S**.

**DEM-H_2_S**, (**P-26**) on the other hand, is a near-infrared (NIR) probe based on coumarin-dicyanoisophorone fluorophore coupled with NBD (7-nitro-1,2,3-benzoxadiazole) as a selective H_2_S recognition group [171]. NBD acts as a quencher, blocking the ICT process, while after reaction with H_2_S, nucleophilic reduction of NBD occurs, leading to the recovery of charge transfer and strong emission in the NIR region (λ_em_ ≈ 710 nm) with a very large Stokes shift (approx. 205 nm), Scheme 14. **DEM-H_2_S** is characterized by high sensitivity (LOD = 80 nM), excellent selectivity, and low cytotoxicity, which has enabled the authors to use it in biological and environmental systems. The **DEM-H_2_S** probe was used to determine H_2_S in water and food samples, as well as to image exogenous and endogenous H_2_S in MCF-7 cells (human breast adenocarcinoma cell line) and live zebrafish larvae. With emission in the NIR range, a large Stokes shift, and an excellent turn-on signal, **DEM-H_2_S** is a unique tool for the analytical detection of H_2_S in environmental samples and in physiological and pathological studies.

#### 4.1.5. Neurotransmitter Detection

Glutamate is an important excitatory neurotransmitter, while GABA (gamma-aminobutyric acid) is an inhibitory neurotransmitter that reduces the excitability of nerve cells in the brain and spinal cord. These compounds regulate brain function and influence memory, learning, and thought processes [172,173,174]. Dopamine (DOPA), on the other hand, is an essential neurotransmitter that plays a key role in cognitive processes, motivation, and motor control. Abnormal DOPA levels are associated with a number of neurological and mental disorders, such as Parkinson’s disease, schizophrenia, and depression [175,176,177,178]. Detecting imbalances in neurotransmitter levels is important for diagnosing the early stages of diseases related to the nervous system.

**Scheme 15:**
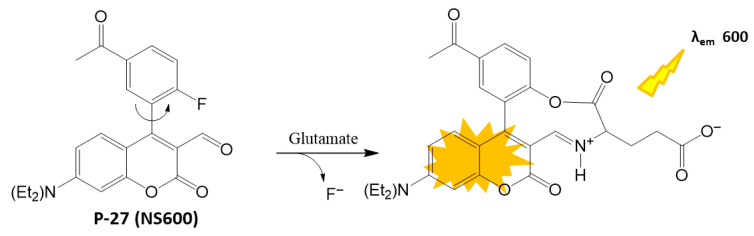
The sensing mechanism of glutamate detection with probe **NS600**.

In 2025, Xu et al. presented a probe designed to detect glutamate and GABA in neurons [179]. The design of the **NS600** (**P-27**) probe is based on the structure of coumarin, in which there is an aldehyde group at the C-3 position and an electron-withdrawing group with a fluorobenzene motif at the C-4 position. This sensor offers a dual-site binding mechanism via an uncommon nucleophilic aromatic substitution reaction in which the nucleophile is a carboxyl group (Scheme 15). Binding to glutamate is accompanied by an increase in fluorescence quantum yield from Φ = 0.0096 (free probe) to Φ = 0.023 for the **NS600**–glutamate adduct. A 270-fold increase in fluorescence is observed with the titration of glutamate and a 220-fold increase with GABA.

The sensing mechanism was elucidated by NMR, mass spectrometry, UV−vis absorbance, and fluorescence techniques. After glutamate binding, the fluorescence signal increases up to 270-fold, and in the case of GABA, approximately 220-fold, with an emission of about λ_em_ = 600 nm, making it one of the most sensitive fluorescence probes to date. Confocal imaging of live rat hippocampus neurons demonstrates that **NS600** is a dependable and effective sensor device for seeing neurotransmitters.

**Scheme 16:**
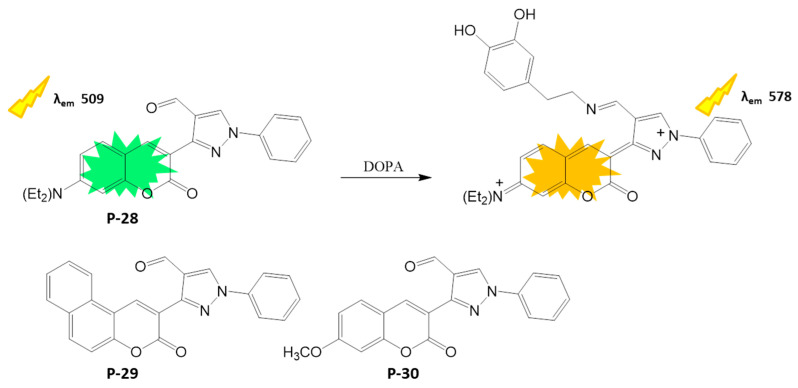
Ratiometric fluorophores probe for DOPA detection.

Three coumarin-based ratiometric fluorophores (**P-28–P-30**) were designed to detect dopamine under conditions of stress and addiction in HEK293 cells (these cells synthesize the neurotransmitter), Scheme 16, [180]. The molecules featured pyrazole-4-carboxaldehyde as the analyte-recognizing group. Among the presented fluorophores, **P-28** proved to be the most promising for neurotransmitter detection. After reacting with dopamine, the charge transfer (ICT) in the probe increases, resulting in a *turn-on* effect (λ_em_ = 578) and an increase in quantum yield. Accordingly, the fluorescence quantum yield of probe **P-28** increases from Φ = 0.051 to Φ = 0.127 after dopamine binding. The authors used the probe for the determination of DOPA in biological fluids and artificial biological fluids with a detection limit of 8.9 nM, which is consistent with physiological dopamine levels. **P-28** in zebrafish larvae is located in the brain region and enables visualization of endogenous dopamine there.

#### 4.1.6. Detection of Other Small Molecules

Among biologically important small molecules, particular attention should be paid to gasotransmitters and reactive sulfur molecules other than those described above, such as SO_2_, CO, and hydrazine. They often play a signaling role and modulate the functions of cellular organelles, and are associated with oxidative stress, vasodilation, energy metabolism, and cell membrane depolarization. Disorders in the levels of these small molecules can result in serious diseases, such as cardiovascular disease, neurodegeneration, stroke, diabetes, and cancer [181,182,183,184]. Recently, a few examples of coumarin-based probes have appeared for these biomarkers.

**Scheme 17:**
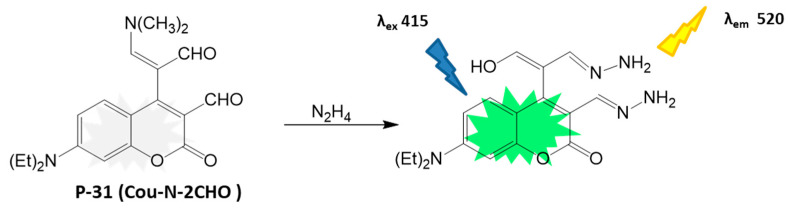
Detection of N_2_H_4_ with probe **Cou-N-2CHO** (**P-31**).

The **Cou-N-2CHO** (**P-31**) probe presented by Liu et al. is a new, highly sensitive coumarin derivative for N_2_H_4_ detection bearing two aldehyde groups [185]. The presence of these groups enables a selective nucleophilic reaction with hydrazine (N_2_H_4_), leading to the formation of an adduct with an intense 22-fold increase in fluorescence (λ_em_ = 520 nm), Scheme 17. The authors confirmed the mechanism of the probe’s reaction with the analyte using spectroscopic techniques (NMR and ESI-MS). **Cou-N-2CHO** exhibits high sensitivity (LOD = 1.02 µM), very good selectivity, and resistance to interference, which allows it to be used even in complex environmental matrices. The coumarin derivative **Cou-N-2CHO** has been used for imaging hydrazine in living cells and for soil analysis.

**Scheme 18:**
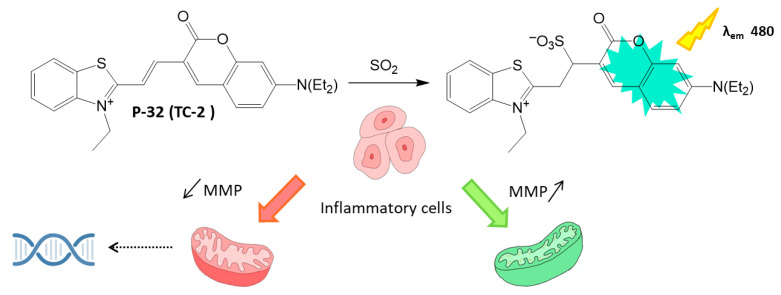
The study of mitochondrial dysfunction with probe **TC-2**.

The **TC-2** (**P-32**) probe, on the contrary, is a dual-function ratiometric fluorophore obtained by combining the coumarin and thiazolium skeletons [186]. Research has shown that this probe can be used for the simultaneous detection of mitochondrial SO_2_ and changes in mitochondrial membrane potential (MMP), Scheme 18. In addition, **TC-2** has the ability to bind specifically to DNA. In normal mitochondria, when MMP is high, **TC-2** remains in the mitochondria and enables SO_2_ detection. The presence of the analyte induces a nucleophilic addition reaction to the C=C bond, which further leads to the formation of a form that emits shorter wavelength signals. **TC-2** shows a decrease in emission at 665 nm and a simultaneous increase in intensity at ~480 nm, enabling ratio-metric detection with a very low limit of detection (LOD = 13.8 nM). Probe **TC-2**, fluorescence quantum yield shows a strong dependence on solvent polarity and excitation wavelength, reaching Φ = 38.27% in 1,4-dioxane at λ_ex_ = 400 nm, while remaining moderate in DMSO (Φ = 4.69% at λ_ex_ = 400 nm and Φ = 30.73% at λ_ex_ = 525 nm). When mitochondria are damaged, MMP decreases, and the probe is no longer retained in this organelle and migrates to the cell nucleus, where it starts to bond to DNA. This results in an increase in fluorescence intensity (λ_ex_ = 561; λ_em_ = 665) and an increase in fluorescence lifetime. **TC-2** has a structure that allows electrostatic interaction with the anionic DNA backbone and partial intercalation (due to the planar conjugated fragment).

The **TC-2** probe has a very practical application, allowing simultaneous imaging of SO_2_ levels and MMP changes in living HepG2 cells. In addition, the possibility of monitoring changes in LPS-induced inflammation models in vitro and in vivo (in mice) in the study of mitochondrial dysfunction and oxidative stress was presented.

**Scheme 19:**
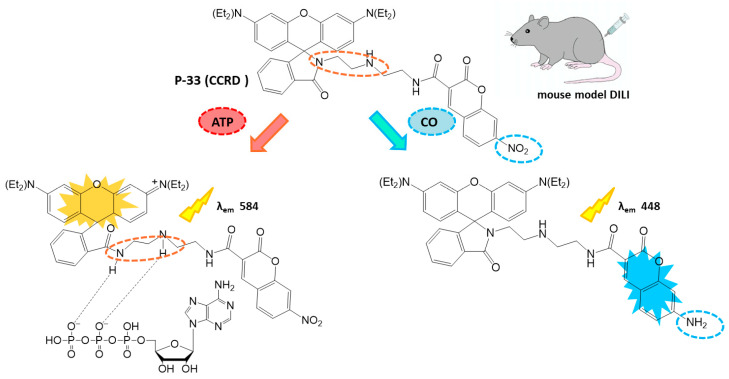
**CCRD** probe operating scheme; simultaneous detection of mitochondrial CO and ATP.

The **CCRD** (**P-33**) probe is a dual-function fluorescent molecule that enables simultaneous monitoring of mitochondrial CO and ATP in two completely separate emission channels [187], Scheme 19. The structure of the molecule consists of a nitro-coumarin derivative, which is the CO-recognizing fragment, and a rhodamine derivative coupled with a triamine chain, which is responsible for ATP detection.

The reduction of the NO_2_ group by CO to a free amine unblocks the PET process and causes intense blue emission (λ_em_ = 448 nm) from coumarin. In turn, the coordination of ATP to diethylenetriamine opens the spirolactam ring of rhodamine, inducing a strong red signal (λ_em_ = 584 nm). The **CCRD** probe is characterized by high selectivity and low detection limits (LOD CO = 38 nM; LOD ATP = 5 μM), is effectively targeted to mitochondria, and has very low cytotoxicity. **CCRD** enables imaging of endogenous and exogenous CO/ATP changes in HepG2 cells and in the liver of mice with *Drug-Induced Liver Injury* (DILI). It allows monitoring of CO and ATP signal changes in mitochondria during drug-induced hepatocyte damage, thereby making the probe a powerful tool for redox research, mitochondrial metabolism, and early diagnosis of DILI.

The Velmathi group has developed an **NCrHT** (**P-34**) probe for the detection of sulfur mustard (2,2′-dichloro-diethyl sulfide) [188]. Probes of this type are rare, and there are few studies undertaking the detection of this analyte [189,190,191,192]. Sulfur mustard is a chemical warfare agent; the authors used its simulant, 2-chloroethylsulfide (CEES).

**Scheme 20:**
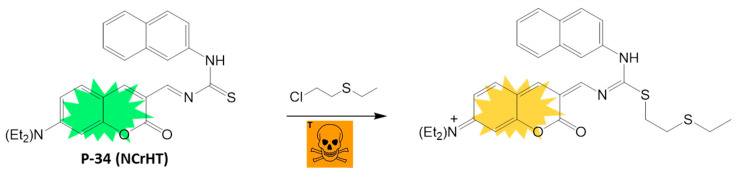
**NCrHT** probe for detection 2-chloroethylsulfide (CEES).

The probe operates on the ICT-off mechanism. In solution, it exhibits intense green emission (λ_em_ = 532 nm), which, after reaction with CEES, shifts to 575 nm and undergoes strong quenching (response time <5 s), Scheme 20. The probe exhibits a moderate fluorescence quantum yield (Φ = 0.105), which markedly decreases to 0.0225 upon reaction with CEES. **NCrHT** was used in RAW 264.7 cells (mouse macrophages) to allow imaging of the degree of CEES poisoning, and in vivo studies on zebrafish demonstrated both the ability to detect CEES in the brain and to monitor detoxification using the lipophilic scavenger *N*-acetylcysteine, which restores the fluorescence of the probe. **NCrHT** is therefore a rare example of a fluorescent probe capable of detecting chemical warfare alkylating toxins and observing detoxification processes in vivo.

### 4.2. Probes for Reactive Oxygen and Nitrogen Species

Hydroxyl radical (•OH), hypochlorite (ClO^−^), peroxynitrite (ONOO^−^), and H_2_O_2_ are examples of reactive oxygen species (ROS) and reactive nitrogen species (RNS) that have strong oxidizing properties and can directly oxidize proteins, nucleic acids, and lipids, causing irreversible cell damage and serious diseases [193,194,195,196,197,198,199,200]. The use of highly sensitive and selective methods for real-time ROS monitoring allows for a better understanding of their role in pathological processes.

Probes dedicated to ROS and RNS require high selectivity and stability due to the fact that in real conditions there are many simultaneous reactive species. To overcome this challenge, mechanisms based on the chemical reaction of the probe with a specific analyte are preferred. The crucial approach is to employ classic fluorescence mechanisms such as ICT, ESIPT, PET, or FRET and to design a suitable donor/acceptor group that allows for the modulation of emission after reaction with ROS/RNS. For example, fluorophore masking with phenolic, thioether, or hydrazone groups is often used, which, after selective oxidation, transforms into stronger electron donors or acceptors, generating a turn-on signal [201,202,203,204].

#### 4.2.1. HClO Detection

An example of dual-module probes for selective HOCl detection are **PMF** (**P-35-37**) probes [205]. The probes were designed by Zhang’s group and feature a phenothiazine-coumarin structure (responsible for the ratiometric response) and a leuco methylene moiety (NIR-turn-on module) connected to each other by an aliphatic diamine with a different chain length. The probes have two independent reaction sites with the analyte: sulfur in phenothiazine and leuco-MB, which is a reduced form of methylene blue dye. The fluorescence quantum yields of the probes **P-35**, **P-36**, and **P-37** were reported to be low but measurable, with Φ values of 1.5%, 2.1%, and 2.4%, respectively, in the test medium.

**Scheme 21:**
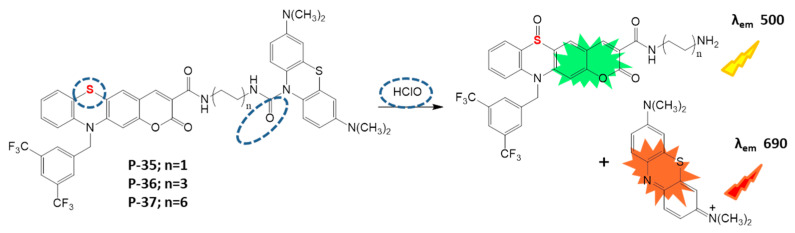
Highly photostable HClO probe.

For application testing, a probe with the longest diamino-linker, **P-37**, was selected, which ensured better separation of the phenothiazine-coumarin and leuco methylene moiety modules. In the presence of HClO, both reactive sites in **P-37** oxidize, generating two types of signals: emission shifts from 630 to 500 nm (ratiometrically) and strong NIR emission enhancement at 690 nm, Scheme 21. The sensitivity (LOD = 19.2 nM) and selectivity of the probe are remarkably high. Furthermore, **P-37** does not respond to other ROS, amino acids, or metal ions. Due to its lipophilic group, PMF3 also demonstrated a strong affinity for lipid droplets, which was significant in its application to DILI imaging (a model of liver damage caused by paracetamol).

The **PBFF** (**P-38**) probe is another probe that features a phenothiazine-coumarin motif as the HClO reaction site (oxidation of S to SO) [206]. A large lipophilic 3,5-bis(trifluoromethyl)phenyl group has been additionally incorporated into the molecule structure, which acts as a rotor; the free rotation is prevented, forcing the fluorophore to aggregate in an aqueous solution. The lack of rotation blocks TICT (*Twisted Intramolecular Charge Transfer*). TICT is a non-emissive excited state that results from rotation between the donor and acceptor modules, leading to fluorescence quenching.

**Scheme 22:**
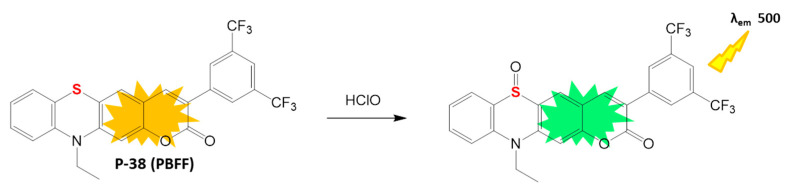
Mechanism of action of the ratiometric PBFF probe in response to HClO.

The **PBFF** probe is ratiometric and functions on a single fluorophore. Blocking TICT induces strong emission in the initial state and a clear shift in emission from 596 nm to 500 nm after sulfur oxidation (Scheme 22). The probe is very selective, sensitive (LOD 15.7 nM), and good at imaging changes in HClO in living cells when they are under oxidative stress and an inflammatory response. The probe shows a relatively high fluorescence quantum yield in aqueous media (Φ ≈ 0.20), which increases upon oxidation. **PBFF** also allowed for detailed mapping of HOCl distribution in an isoniazid-induced liver injury model, revealing differences between healthy and damaged tissues. Furthermore, **PBFF** has been successfully used to image HOCl in vivo in whole organs and tissue sections, confirming its usefulness in monitoring pathological oxidative stress in the context of liver disease.

**Scheme 23:**
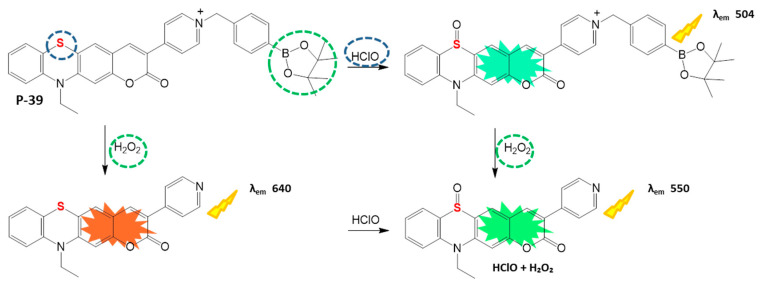
Multicolor imaging of HClO and H_2_O_2_ with **P-39**.

In another probe **P-39** with a phenothiazine-coumarin motif, a boronate group was additionally used, which reacts specifically with H_2_O_2_ [207]. Sulfur oxidation generates a green signal (λ_em_ = 504 nm), and boronate cleavage generates a red signal (λ_em_ = 640 nm). In the case of a mixture of HClO + H_2_O_2_, the simultaneous excitation of both mechanisms leads to yellow emission (λ_em_ = 550 nm), Scheme 23. The research revealed the inaugural use of a singular fluorescent probe for real-time, multicolor imaging of both endogenously and exogenously generated HClO and H_2_O_2_ within the mitochondria of living cells and zebrafish.

**Figure 21:**
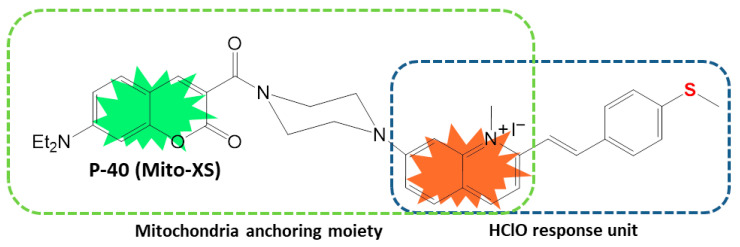
Structure of multifunctional single-molecular fluorescent probe **Mito-XS**.

Another probe **Mito-XS** (**P-40**) employs a “three-in-one” strategy, having designed and developed a multifunctional single-molecular fluorescent probe **Mito-XS** that facilitates ratiometric, specific, and sensitive detection of ClO^−^, while also enabling firm anchoring in mitochondria and precise analysis of mitochondrial morphology [208]. The probe is composed of quinoline salt and alkyl-aryl thioether, which serve as ClO^−^ response units, and 7-diethylaminocoumarin moiety, which acts as an internal reference for ratiometric detection of ClO− and is linked by piperazine. A probe designed in this way produces two emissions separated by 125 nm: stable green fluorescence at ~500 nm and a red signal at ~625 nm susceptible to oxidation by ClO^−^ (Figure 21). Oxidation of the thioether group by ClO^−^ causes a strong reduction in red emission, enabling highly sensitive detection (LOD = 18 nM) and a fast signal (<1 min). This process is accompanied by a moderate change in fluorescence efficiency, with the quantum yield decreasing from Φ = 5.40% for the native probe to Φ = 3.57% after oxidation.

**Mito-XS** allowed for real-time monitoring of mitochondrial morphology and ClO^−^ fluctuation during oxidative stress. It was used to evaluate the therapeutic effects of drugs in CCl_4_-treated HepG2 cells. In in vivo applications, **Mito-XS** provides dual-color imaging of liver fibrosis in mice and assessment of the efficacy of treatments involving drugs such as silymarin, methylated ferulic acid, and puerarin. This positions the probe molecule as an advanced tool for studying the pathophysiology of fibrosis, oxidative stress, and drug efficacy assessment.

The super-resolution **SFL-HClO** (**P-41**) probe was developed as a tool for imaging HClO changes in the atherosclerosis process and for analyzing the formation of foam cells, which are an early marker in the development of atherosclerotic plaques, Figure 20 [209]. In vivo, the probe allowed the detection of high HClO concentrations within the carotid artery in ApoE^−/−^ mice. It can therefore be a diagnostic tool for assessing early oxidative stress in atherosclerotic plaques. In turn, in in vitro models, the probe allows for the analysis of processes related to lipid deposition and simultaneous imaging of lipid droplets (LDs) and the local presence of HClO in RAW 264.7 macrophages treated with Ox-LDL (oxidized low-density lipoprotein). The **SFL-HClO** probe is ratiometric and consists of a spirolactone skeleton in which the carbonyl group is protected by a thioether fragment. Before reacting with HClO, **SFL-HClO** emits intense green fluorescence (λ_em_ approx. 579 nm). After reacting with the analyte, the C–S bond breaks and the fluorophore is released (λ_em_ approx. 642 nm).

**Figure 22:**
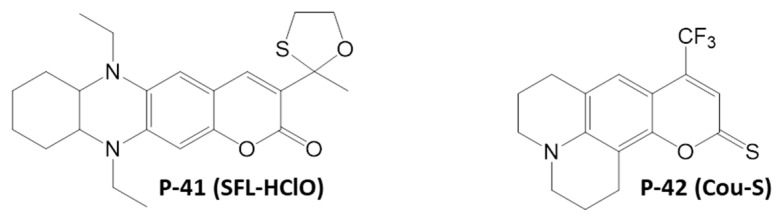
Probes **P-41** and **P-42**.

The **Cou-S** (**P-42**) thiocoumarin probe was prepared from a 4-CF_3_-coumarin derivative in a simple reaction with Lawson’s reagent, Figure 22 [210]. The –C(S)– groups are reactive sites for HClO and quench emission through a strong PET mechanism. After oxidation by HClO, the thioester is converted to the corresponding derivative, which blocks PET and produces intense green fluorescence with a maximum at 540 nm upon excitation at 465 nm. For the hypochlorite-responsive probe **Cou-S**, a very low fluorescence quantum yield (Φ_F = 0.005) was reported, which increased dramatically to Φ_F = 0.595 upon reaction with analyte. **Cou-S** has a rapid response time (3 s at 50 µM HClO), high sensitivity (LOD = 15.6 nM), and excellent selectivity over other ROS and biothiols. **Cou-S** has been employed to detect both exogenous and endogenous HClO in HeLa and RAW 264.7 cells. In vivo studies have been performed to image cisplatin-induced acute kidney injury in live mice. After intravenous administration of **Cou-S**, a strong fluorescent signal appears selectively in the kidneys of mice, while in healthy animals, the emission is minimal. Additional histological tests and cryopreservation imaging confirmed the possibility of early diagnosis of renal failure by detecting HClO generated as a result of oxidative stress.

**Figure 23:**
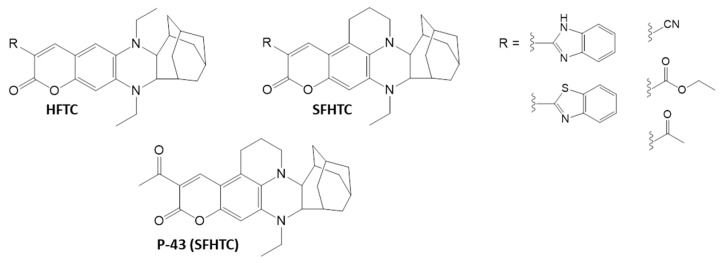
Chemical structures of coumarin-designed SHFTC and HFTC probes.

Recently, Liu et al. presented a series of ratiometric fluorescent dyes (**SFHTC** and **HFTC**) for HClO detection, with the side-chain-fixed homoadamantane-fused tetrahydroquinoxaline as the electron-donating group, Figure 23 [211]. Among the dyes, probe **P-43** was selected, which was superior to other analogues in terms of photostability, signal brightness, large Stokes shift (≈160–180 nm), and ICT switching efficiency. After the reaction with HClO, the probe undergoes oxidative desulfurization, which exposes the acceptor group and restores intense red emission (≈650–700 nm). This transformation is accompanied by an increase in fluorescence efficiency, with the quantum yield of the resulting probe reported as Φ = 0.07 in PBS/CH_3_CN (7:3, pH 7.4). Specifically for **SFHTC** (**P-43**), its absorbance may be maintained at over 93% after 60 min of continuous irradiation with a laser source. **SFHTC P43** demonstrated superior sensing capabilities for HClO in vitro and successfully achieved HClO imaging in live cells. The application of probe for prolonged continuous imaging of HClO variations in both HeLa cells and the animal model was successfully achieved.

#### 4.2.2. H_2_O_2_ Detection

**Figure 24:**
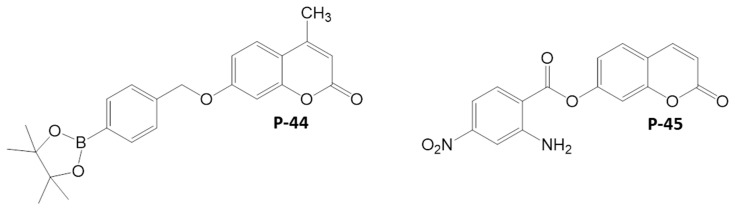
Selective probes for detection of H_2_O_2_.

Recently, two H_2_O_2_ detection probes with a similar mechanism of action have been presented, in which the ester/ether group undergoes specific cleavage in the presence of H_2_O_2_, leading to the release of emission (Figure 24).

The **P-44** probe has boronate esters as a recognition unit [212]. After binding the analyte, the borate ester group of **P-44** is selectively oxidized and hydrolyzed by the target H_2_O_2_. The probe exhibits a characteristic spectral change: the absorption maximum shifts from 321 nm to 372 nm, while the fluorescence maximum appears at 451 nm. The probe has been used in biological studies to image both exogenous and endogenous H_2_O_2_. It enables the visualization of oxidative stress in RAW264.7 and SW480 cells. In turn, the **P-45** probe, in the presence of the analyte and the release of 7-hydroxycoumarin, provides a rapid increase in emission at 455 nm [213]. The probe demonstrated substantial fluorescence enhancement and excellent selectivity (LOD = 0.18 μM). It was then employed to accurately detect H_2_O_2_ in biological systems and physiological contexts.

**Figure 25:**
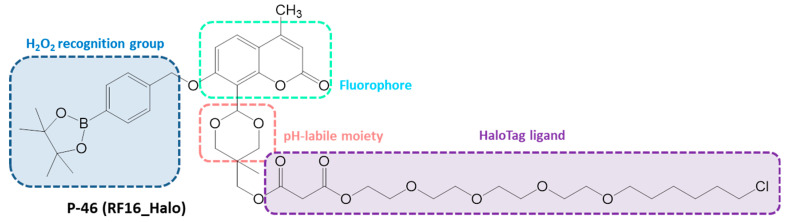
Structure of the macromolecular coumarin probe **RF16_Halo**.

In 2024, Shao presented a novel approach to synthesize the fluorescent macromolecular compound **RF16_Halo** (**P-46**) [214]. **RF16** was initially synthesized by connecting an H_2_O_2_-sensitive boron cage to the C-7 OH group and a HaloTag ligand to the pH-sensitive 1,3-dioxane group of the coumarin fluorophore (Figure 25). In the **RF16** structure, the coumarin core is responsible for fluorescence, the boronic ester recognizes H_2_O_2_, the 1,3-dioxane acetal at position C-8 is pH-labile and hydrolyzes in an acidic environment, while the HaloTag ligand enables specific binding to the protein. The probe enables precise imaging of H_2_O_2_ in a defined location within the cell (first binding covalently to a protein) and only under dual control; fluorescence appears after hydrolysis of the acetal ring in an acidic microenvironment and after the reaction of boronate with H_2_O_2_. The **RF16** probe demonstrated considerable fluorescence enhancement and great selectivity (LOD = 0.18 μM). The authors used the probe to observe dynamic changes in H_2_O_2_ levels in living cells and to track oxidative activity in signalling pathways, especially during chemically induced oxidative stress.

#### 4.2.3. HNO Detection

Nitroxyl (HNO) is a single-electron reduced form of nitric oxide (NO) and plays an important role in physiological and pathological processes. Despite its similar structure to NO, HNO displays different chemical and biological properties. HNO can inhibit aldehyde dehydrogenase activity by interacting with protein thiols, which is important in the treatment of alcoholism and in protecting tissues from ischemic damage. Recent studies also indicate that HNO level disorders promote the development of diseases such as inflammation, liver damage, and cancer [215,216,217]. Effective methods for detecting this analyte, both in vitro and in vivo, are highly needed.

**Scheme 24:**
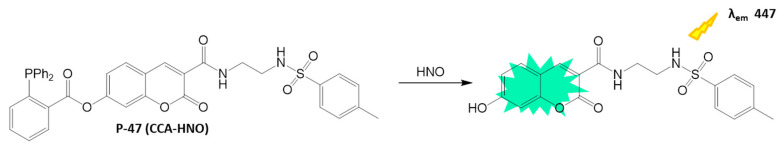
Reaction mechanism of the coumarin-based probe **CCA-HNO**.

Recently, a probe designed for the selective detection of HNO in the presence of metal ions, anions, biothiols, and reactive oxygen species has attracted attention [218]. The **CCA-HNO** (**P-47**) probe was obtained through the esterification of the hydroxyl group on the coumarin structure using 2-(diphenylphosphonyl)benzoate as a distinctive identifying moiety for HNO. The analyte reacts with phosphine, initiating the cleavage of the phosphine group and releasing a 7-hydroxycoumarin derivative (λ_em_ = 447 nm), Scheme 24. Furthermore, the **CCA-HNO** probe demonstrated a low detection limit of 34 nM and a wide pH range of 2 to 12 during HNO sensing. The coumarin derivative was effectively utilized for imaging nitroxyl in HeLa cells and for tracking its endogenous and exogenous synthesis. Additionally, the probe enabled visualization of changes in HNO levels in zebrafish, confirming its usefulness in biological studies on nitrosative stress and diseases associated with HNO dysregulation.

#### 4.2.4. ONOO^−^ Detection

Peroxynitrite (ONOO^−^) is a reactive oxygen species (ROS) often produced when superoxide (O_2_•^−^) interacts with nitric oxide (NO). ONOO^−^ has been observed to facilitate oxidative damage and induce lipid peroxidation, thus triggering ferroptosis. The alterations in ROS will consequently be accurately represented by dependable ONOO^−^ detection, which may greatly assist in clarifying its biological implications during ferroptosis and further facilitate comprehensive examination and modulation of ferroptosis [219,220].

**Figure 26:**
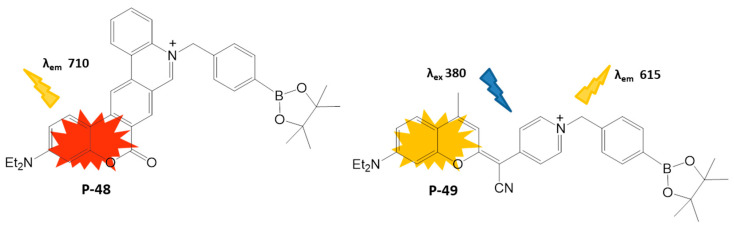
The coumarin-based probes toward peroxynitrite (ONOO^−^).

Two highly selective probes for ONOO^−^ detection with strong near-infrared emission were presented by James’ group (Figure 26). Probe **P-48** was created by combining a coumarin skeleton and phenanthridine [221]; probe **P-49** [222] is a coumarin-pyridinium dye (COUPY) with a D-π-A type fluorophore developed by Marchán’s group [223]. Probe **P-48** emits strong NIR λ_em_ = 710 nm (with a large Stokes shift of 200 nm), which is completely quenched after reaction with ONOO^−^ with a boronate group. Probe **P-49**, on the other hand, is ratiometric with high selectivity and low toxicity. The dihydropyrimidine group is the main site of reaction with HNO, followed by emission at 447 nm. **P-48** and **P-49** were used to track changes in ONOO^−^ concentrations in living cells, zebrafish, mice, and *Arabidopsis thaliana*.

**Scheme 25:**
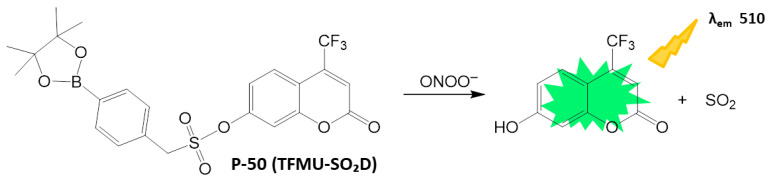
The ONOO^−^ triggered fluorescence SO_2_ donor probe.

The unusual ONOO^−^ triggered fluorescence SO_2_ donor probe, **TFMU-SO_2_D** (**P-50**), was developed by Liu et al. [224]. The molecule contains the ONOO^−^-responsive aryl boronate moiety and the fluorophore of 7-hydroxy-4-(trifluoromethyl) coumarin with sulfonates. The **TFMU-SO_2_D** was used for highly accurate monitoring of peroxynitrite in the process of ferroptosis in vitro and in vivo. Upon activation by ONOO^−^, the probe emits a strong fluorescent signal (λ_em_ = 510 nm) and simultaneously releases SO_2_, which compensates for the decrease in ROS caused by conventional probes (Scheme 25).

In HeLa, HepG2, and RAW 264.7 cells, **TFMU-SO_2_D** was utilized to detect ONOO^−^ and facilitate SO_2_ release beneath oxidative stress induced by monensin for the inaugural instance. The ONOO^−^ flux and SO_2_ emission during erastin- and RSL3-induced ferroptosis were elucidated. Following the validation of **TFMU-SO_2_D**’s capacity to evaluate the ferroptosis process via ONOO^−^ imaging, an additional inquiry was conducted to determine whether SO_2_ emitted by TFMU-SO_2_D might mitigate the depletion of ROS.

**Scheme 26:**
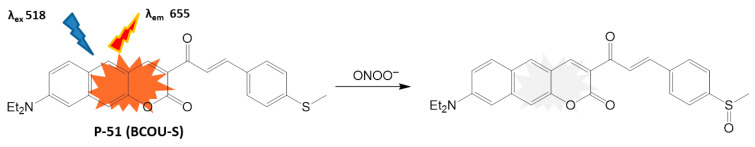
Detection mechanism of **BCOU-S** for ONOO^−^.

The combination of a coumarin core extended in the π direction with a methyl thioether group as the ONOO^−^ recognition group resulted in the formation of the **BCOU-S** (**P-51**) molecule [225]. It is a fluorescent NIR probe (λ_em_ = 655 nm; λ_ex_ = 518 nm), in which the methyl thioether group is the site of reaction with ONOO^−^ and undergoes oxidation, leading to strong fluorescence quenching (Scheme 26). This oxidation process is accompanied by a dramatic decrease in fluorescence efficiency, with the quantum yield dropping from 38.8% for BCOU-S to 0.31% after reaction with ONOO^−^. The probe was used to simultaneously monitor two independent parameters relevant for the early detection of drug-induced liver injury (DILI): ONOO^−^ levels and the state of lipid droplets (LDs). The effectiveness of **BCOU-S** was demonstrated in models of induced hepatotoxicity, where the probe allowed tracking of ONOO^−^ increase and dynamic changes in LDs.

**Scheme 27:**
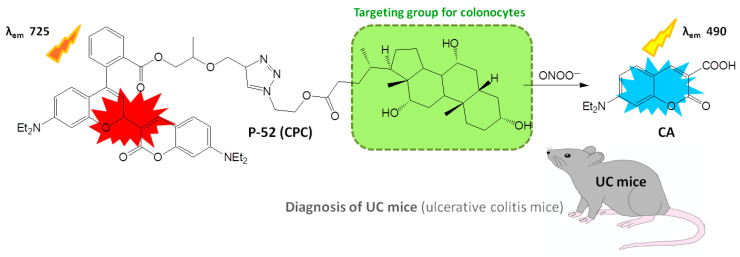
Colon targeting fluorescence probe **CPC**.

The **CPC** (**P-52**) probe is the first ratiometric ONOO^−^ sensor designed specifically to localize in the colon [226]. It was created by combining a pyran-coumarin fluorophore with cholic acid. Cholic acid recognizes the TGR5 receptor in colonocytes. The probe exhibits an enormous emission shift of 235 nm, transitioning from strong NIR emission (λ_em_ = 725 nm) to distinct blue emission (λ_em_ =490 nm) upon analyte binding (Scheme 27).

The mechanism of action is based on the oxidative cleavage of part of the oxonium by ONOO^−^ and the formation of a weakly coupled **CA** fluorophore. CPC has been used in studies on cells, in vitro inflammation models, and mice. It allowed imaging of both exogenous and endogenous ONOO^−^ in NCM-460 cells (normal human colon mucosal epithelial cells), as well as differentiating healthy cells from cells with high levels of endogenous ONOO^−^ (LPS/IFN-γ stimulated; inflammation model). Undoubtedly, the **CPC** probe is an extremely valuable diagnostic tool, combining ratiometric detection of ONOO^−^ with selective targeting of the large intestine.

### 4.3. Probes for Detection of Anions and Cations

The selective and sensitive detection for trace transition metal ions as well as anions has received great attention in biological samples. Metal detection is particularly important in biology, clinical medicine, environmental chemistry, and food quality control. In cells, metal ions are active in the transport of electrons and small molecules in living organisms and also function as Lewis acids and catalysts for redox reactions. Essential inorganic ions such as Na^+^, K^+^, Ca^2+^, Mg^2+^, Zn^2+^, and Fe^2+^/Fe^3+^ regulate numerous biological processes, including intracellular and intercellular signaling, DNA transcription, proper neuron function, oxygen transport, photosynthesis, and many electron transfer reactions.

On the other hand, non-essential or toxic metal ions (Cd^2+^, Hg^2+^, Pb^2+^, As^3+^, Cr^3+^/Cr^6+^, Co^2+^, Ni^2+^, and others) are mainly produced as a result of human activity, including mining, industrial wastewater discharge, and fossil fuel combustion. These elements accumulate in ecosystems and the food chain, leading to serious environmental and health risks. It is worth noting that even essential ions can be toxic if their concentration exceeds physiological norms. Fluorescent probes appear to be an ideal tool for monitoring ions in biological, chemical, and environmental systems, as well as for real-time monitoring of molecular processes and detection of even trace amounts of analytes [179,227,228,229,230,231,232]. Depending on the designed system, the detection of these analytes is based on basic photophysical mechanisms: fluorescence resonance energy transfer (FRET), intramolecular charge transfer (ICT/CT), chelation-enhanced fluorescence (CHEF), photoelectron transfer (PET), or the formation of excimers and excimers. Each of these processes results in characteristic changes in the absorption or emission spectrum of the probe, enabling selective recognition of specific metal ions and biologically and environmentally relevant anions (e.g., CN^−^, SO_3_^2−^).

#### 4.3.1. Anions Detection

Sulfite (SO_3_^2−^) and bisulfite (HSO_3_^−^) anions are produced in industrial processes and are the result of the metabolism of sulfur amino acids. They serve specific physiological functions in cells, but in excess they can be toxic. High concentrations of these anions increase cell membrane permeability, disrupt enzyme activity, and can lead to diseases of the nervous and cardiovascular systems [233,234,235]. The Golgi apparatus is highly susceptible to redox imbalances and sulfur stress, prompting the invention of certain fluorescent probes with Golgi-targeting moieties to provide selective imaging of localized variations in SO_3_^2−^/HSO_3_^−^ concentration and the resultant alterations in organelle viscosity. The detection of SO_3_^2−^ and HSO_3_^−^ is therefore also important from the perspective of medical diagnostics.

**Scheme 28:**
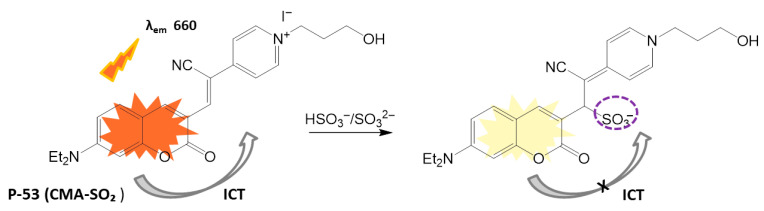
Reaction mechanism of the ratiometric probe **CMA-SO_2_** toward SO_3_^2−^/HSO_3_^−^.

Wang et al. presented the operation of a ratiometric and highly water-soluble **CMA-SO_2_** (**P-53**) probe emitting red light [236]. **CMA-SO_2_** was designed to detect SO_2_ derivatives (SO_3_^2−^/HSO_3_^−^) in aqueous solutions and biological systems. The probe’s structure included a coumarin core, which was responsible for emission in the λ_em_ range of approx. 660 nm, and a malachite green moiety, which extended the π–π system and increased the absorption intensity. The mechanism of action of the probe is based on a Michael addition reaction to the C=C bond, which breaks the conjugated system and blocks ICT. This causes a ratiometric change in the signal (λ_em_ from 660 nm to 570 nm), Scheme 28. The **CMA-SO_2_** probe is characterized by a very low detection limit (LOD 12.56 nM), high selectivity and stability over a wide pH range (2–9), and low cytotoxicity. **CMA-SO_2_** exhibits a fluorescence quantum yield of 0.182 in its native form, which decreases to 0.0343 after reaction with HSO_3_^−^. It has been successfully employed to monitor SO_3_^2−^/HSO_3_^−^ in food and water samples, as well as in living cells of three colon cancer cell lines: HCT116, HT-29, and CT-26.

**Scheme 29:**
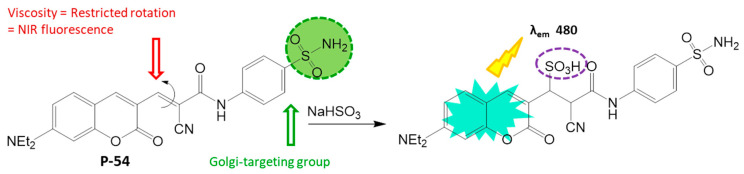
Structure and application of probe **P-55**.

The **P-54** probe was composed of a coumarin skeleton linked to cyanoacetamide. It is a classic donor–π–acceptor (D-π-A) probe, Scheme 29 [237]. The C=C bond in the cyanoacetamide fragment acts both as a reaction site with HSO_3_^−^ and as a molecular rotor responsible for viscosity sensitivity. The molecule also contains a sulfanilamide group, which acts as a specific ligand directing the probe to the Golgi apparatus. After binding, the analyte generates a fluorescent signal at 480 nm (λ_ex_ = 400 nm). Under conditions of increased cell viscosity, intramolecular rotation is inhibited, allowing complete ICT, and the probe then emits light at λ_em_ = 590 nm (λ_ex_ = 500 nm). Both signals are well separated spectrally, enabling simultaneous detection of two parameters. The probe provides simultaneous monitoring of HSO_3_^−^ levels and viscosity changes in the Golgi apparatus. It has been used for imaging in HeLa cells, *Arabidopsis thaliana* tissues, zebrafish (*Danio rerio*), and for determining HSO_3_^−^ in real samples.

**Scheme 30:**
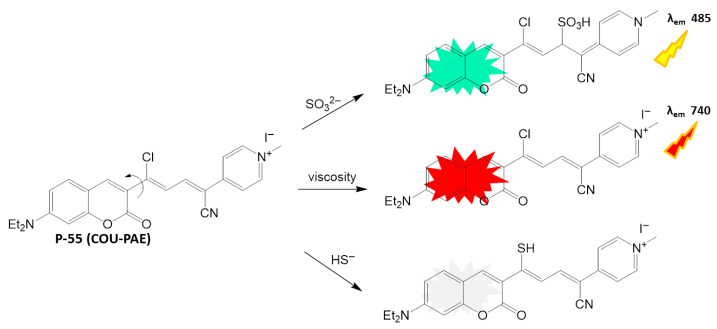
Sensing mechanisms of the multifunctional coumarin-based probe **COU-PAE**.

A multifunctional near-infrared fluorescence probe **COU-PAE** (**P-55**) that can simultaneously detect HS^−^, SO_3_^2−^, and viscosity, which is based on coumarin and responds in different modes to analytes and viscosity was presented by Liu et al. [238]. The probe has three different response mechanisms, Scheme 30. The nucleophilic substitution reaction of the probe with HS^−^ has a turn-off effect—emission at NIR 740 nm rapidly disappears. In turn, SO_3_^2−^, adding to the C=C bond and breaking the coupling, causes a decrease in 740 nm emission and an increase in the signal around 485 nm.

Cell viscosity, on the other hand, is related to the TICT mechanism: twisted intramolecular charge transfer (turn-on); at higher viscosities, the rotation of the bond decreases and the fluorescence signal increases 52-fold, with emission around 735 nm.

**COU-PAE** is readily prepared for the monitoring of alterations in HS^−^, SO_3_^2−^, and viscosity inside mitochondria. Despite the quenching-type reaction of the probe **COU-PAE** to HS^−^, it demonstrated an exceptionally low detection limit (LOD = 0.60 nM). The probe’s response to SO_3_^2−^ is ratio type (LOD = 1.28 μM), which efficiently mitigates external interference. The **COU-PAE** probe has been successfully utilized in live cell imaging such as: HeLa and MHCC-97H.

**Figure 27:**
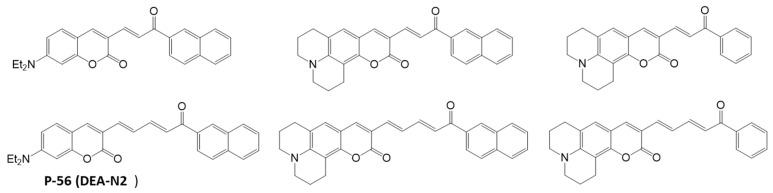
The chemical structures of the probes for SO_3_^2−^ sensitivity.

The work by Yang et al. presents six coumarin-based probes designed to analyze the impact of molecular structure on the ratiometric detection of SO_3_^2−^/HSO_3_^−^, Figure 27 [239]. The influence of steric groups (phenyl and naphthalene) on selectivity, quantum yield, SO_3_^2−^ addition sensitivity, and emission shift was compared. Among the series of coumarins, the **DEA-N2** (**P-56**) molecule presented optimal properties, characterized by the strongest ratiometric emission shift (from 688 nm to 487 nm), the highest sensitivity, and the lowest detection limit (0.31 μM). In the sulfite-sensing series the parent coumarin probes exhibit low quantum yields (Φ = 0.011–0.055), whereas their sulfite addition products show markedly enhanced fluorescence, with Φ increasing up to 0.167 for **DEA-N2** highlighting the beneficial effect of extended conjugation. This compound was then employed for ratiometric imaging of sulfite in HeLa and L929 cells.

#### 4.3.2. Metal Detection

Disruptions in the homeostasis of metal ions, such as Fe^2+^/Fe^3+^, Cu^2+^, Zn^2+^, and Al^3+^, are associated with processes such as oxidative stress, protein aggregation, neurotoxicity, and autophagy disorders. Precise detection and visualization of metallic cations in biological environments are critical for understanding disease mechanisms and early diagnosis. Fluorescent cation probes, due to their high sensitivity, resolution, and non-invasiveness, are currently one of the most important tools.

Coumarin probes are often employed to monitor abnormal metal levels in living cells and tissues. The structure of coumarins is favorable for the design of metal-chelating ligands. What is more, recently presented coumarin probes not only allow for the detection of ions but also exhibit therapeutic synergy, e.g., the ability to remove ROS or inhibit Aβ (amyloid β) aggregation. This may provide scientists with an integrated diagnostic and therapeutic tool for Alzheimer’s disease [240,241].

##### Cu^2+^/Al^3+^ Detection

The **NNH** (**P-57**) probe is an example of a dual-function molecule that can differentiate between Cu^2+^ and H_2_S in different emission channels [242]. **NNH** consists of a coumarin skeleton coupled with a β-diketone coordinating unit and an amino side chain, which provides both affinity for Cu^2+^ and reactivity towards nucleophilic sulfide anions.

**Scheme 31:**
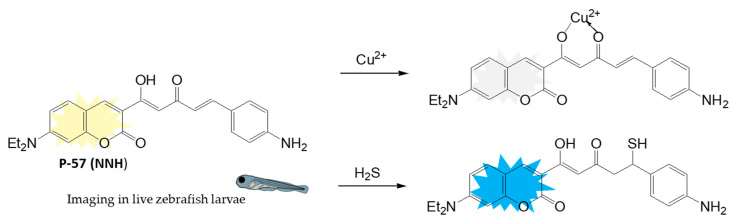
**NNH** probe with tunable blue, yellow and white emissions.

In the presence of Cu^2+^ ions, the probe displays strong fluorescence quenching associated with Cu^2+^ complexation with the β-diketone system. In turn, Michael’s addition of H_2_S to the α,β-unsaturated bond leads to a ratiometric change in emission from 577 nm to 452 nm (Scheme 31). The **NNH** probe allows quantitative detection of both analytes for Cu^2+^ LOD = 0.356 μM and 4.226 μM for H_2_S, with a very fast response (1 min) and high selectivity even in the presence of numerous ionic interferents and biomolecules. NNH enables imaging of changes in Cu^2+^ and H_2_S concentrations in live *Danio rerio* larvae.

**Figure 28:**
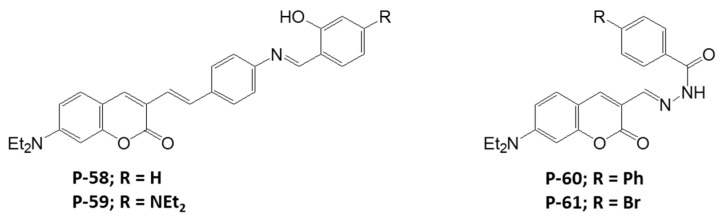
Structure of P-58-61 molecules.

Figure 28 presents Schiff base probes, which operate on the basis of fluorescence turn-off after binding Cu^2+^/Cu^+^. Probes **P-58** and **P-59** are styryl derivatives of coumarin [243]; **P-59** has an additional NEt_2_ group near the copper ion binding site (this enhances the ICT effect). The fluorescence quantum yield was relatively high for the free probes (Φ = 0.83 for probe **P-58** and 0.66 for probe **P-59**) and decreased markedly upon Cu^2+^ binding (Φ = 0.34 and 0.07, respectively). In probes **P-58** and **P-59**, copper ions are complexed with an imine-OH system. **P-59** is characterized by very strong quenching and LOD = 1.56 × 10^−5^ M.

In probes **P-60** and **P-61**, the C=N bond is also the site of copper chelation [244]. These probes initially emit bright green fluorescence (λ_em_ ≈ 525 nm), but after the addition of Cu^2+^ or Cu^+^, the emission is immediately and completely quenched (turn-off). The functionality of the **P-60** and **P-61** probes was further confirmed in imaging in A549 cells and in vivo in zebrafish larvae.

**Scheme 32:**
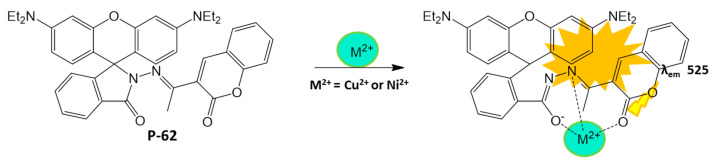
Coumarin-rhodamine probe for metal detection.

The combination of a coumarin skeleton and rhodamine resulted in the development of a fluorometric and colorimetric FRET probe **P-62** for the simultaneous detection of Cu^2+^ and Ni^2+^ ions [245]. Upon metal binding, the spirolactam ring of rhodamine opens, FRET activation occurs, and emission at 580 nm takes place. Cu^2+^ causes a strong and almost complete quenching of the probe signal, while with Ni ^2+^ the quenching is weaker (Scheme 32).

**Scheme 33:**
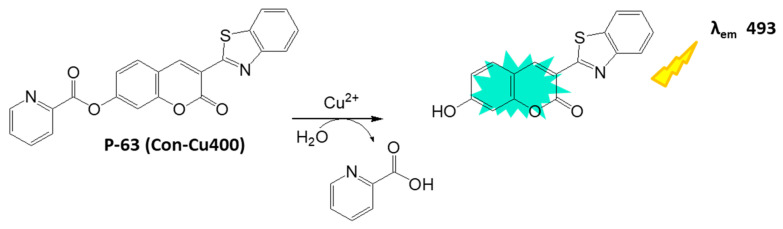
Detection of Cu^2+^ with **Con-Cu400**.

In turn, the **Con-Cu** (**P-63**) probe for detecting Cu^2+^ ions after analyte binding exhibited strong fluorescence (λ_em_ = 493 nm; λ_ex_ = 450 nm), high selectivity, and sensitivity, Scheme 33 [246]. This fluorescence enhancement is accompanied by a marked increase in quantum yield, rising from Φ = 0.081 for the free probe to Φ = 0.36 upon Cu^2+^ coordination. **Con-Cu400** also showed good biocompatibility and could therefore be employed for the fluorescent detection of Cu in both cell and mouse models under normal, copper-depleted, and copper-overloaded conditions.

This probe effectively detected elevated levels of Cu^2+^ in the liver of mice with Wilson’s disease. Wilson’s disease is a rare, congenital metabolic disorder in which toxic amounts of copper accumulate in the body.

**Scheme 34:**
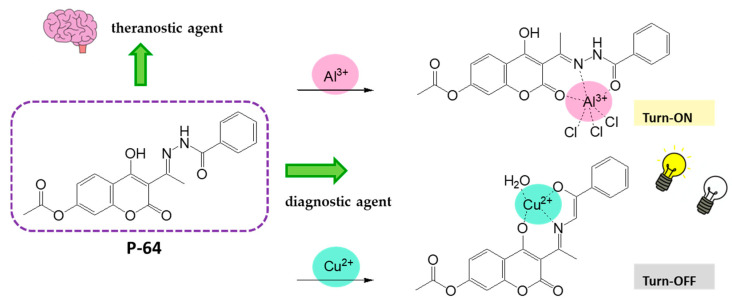
Metal ion-dependent fluorescent theranostic agent **P-64**.

Coumarin compounds with theranostic properties in Alzheimer’s disease have been described only sporadically to date. Yang and colleagues have successfully designed and obtained a hydroxycoumarin Schiff base (**P-64**) as a potential metal ion-dependent fluorescent theranostic agent [247], Scheme 34. Its photophysical properties, possible emission mechanism, and activity against AD-related processes have been analyzed. In addition, two similar monocrystalline forms of 1-Cu(II) complexes were grown, differing in cell parameters, which enabled a detailed assessment of the structure-activity relationship.

The probe **P-64** structure has a coumarin skeleton bearing imine (C-3), hydroxyl (C-4), and carbonyl groups as metal ion chelating sites. After reaction with the Al/Cu analyte, distinct fluorescence on/off reactions were observed, respectively, due to the intramolecular charge transfer (ICT) mechanism. As a result of Al^3+^ chelation, ICT is inhibited and a clear turn-on fluorescence signal with a maximum of 463 nm (λ_ex_ = 370 nm) appears. After Cu^2+^ binding, ICT is enhanced, the electron system is expanded, and fluorescence is strongly quenched (turn-off). The probe displays high selectivity towards Al^3+^ and Cu^2+^ and a low detection limit (LOD ~0.17 µM for Al^3+^ and 0.20 µM for Cu^2+^). The authors also demonstrated that it has the ability to remove reactive oxygen species: in vivo in Caenorhabditis elegans, the probe significantly reduces the level of ROS (to approx. 38% of the control value at 40 µM).

Unlike most classical fluorophores, **P-64** not only detects Al^3+^/Cu^2+^ ions, but also has the ability to actively inhibit spontaneous and induced Aβ aggregation, and its complex with Cu^2+^ exhibits an even stronger inhibitory effect. This combination of diagnostic function and real impact on one of the key processes of AD pathogenesis is rare and is a distinguishing feature of this theranostic probe.

**Scheme 35:**
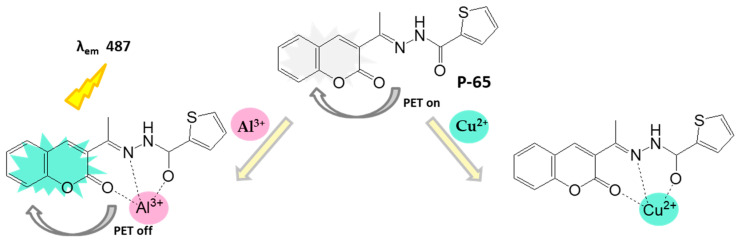
Detection of Al^3+^ and Cu^2+^ with **P-65** probe.

The Zhao group also presented an Al/Cu detection **P-65** [248]. Their probe is supported on a coumarin structure combined with a thiophene-carboxylic hydrazide moiety. The action of **P-65** is based on two different mechanisms: CHEF and inhibition of PET/C=N isomerization in the presence of Al^3+^, which leads to a clear turn-on signal (intense emission at approx. 484 nm), and paramagnetic fluorescence quenching upon Cu^2+^ binding, which causes a strong turn-off effect and a color change of the solution from cyan to colorless, Scheme 35.

The probe exhibits very high sensitivity (LOD: 23.83 nM for Al^3+^ and 12.57 nM for Cu^2+^) and high selectivity confirmed by interference tests. Another important feature is mechanochromism—friction of the powder causes a shift in the emission maximum from 473 to 523 nm, and dichloromethane vapors reverse the effect. The fluorescence efficiency strongly depends on the physical state of the probe, with a low quantum yield in solution (Φ = 0.005) and a much higher value in the solid state (Φ = 0.452). In biological studies (HeLa cell), **P-65** proved to have low cytotoxicity, good cell permeability, and enabled clear visualization of both ions.

##### Hg^2+^ Detection

**Figure 29:**
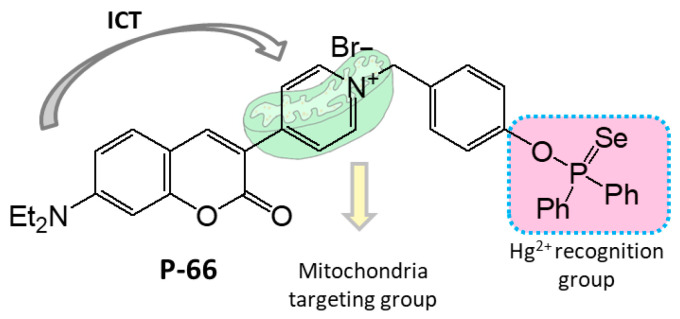
Architecture of probe **P-66**.

A pioneering and highly targeted mitochondrial probe **P-66** for the detection of Hg^2+^ ions was designed by Zhang’s group [249]. The probe consists of a coumarin fluorophore, a pyridine group responsible for targeting mitochondria, and a diphenylphosphinoselenoate recognition group connected by a *p*-hydroxybenzyl bridge (Figure 29).

In the presence of the analyte, a chemical reaction occurs in which mercury ions induce deselenation and P–O bond cleavage, leading to the formation of fluorophore through the elimination of 1,6. This chemical sequence triggers a spectacular photophysical change: a shift in the absorption peak from 492 to 426 nm (Δ66 nm) and a distinct discoloration of the solution (orange → pale yellow); a fluorescence shift from 570 to 498 nm (Δ72–95 nm); a large Stokes shift (78 nm); minimal interference and LOD = 16.7 nM; and a very stable ratiometric signal. Accordingly, the fluorescence quantum yield increases markedly from Φ ≈ 0.29 to Φ ≈ 0.69 upon probe activation. In addition, probe **P-66** is also characterized by exceptional selectivity—it reacts only with Hg^2+^, even in the presence of many other metal ions, and is precisely targeted to mitochondria.

In biological studies, the probe demonstrated very good HeLa cell viability (>92% at 10 µM), excellent cell permeability, and the ability to ratiometrically monitor changes in Hg^2+^ concentration in living cells in two emission channels (green and red). In the presence of Hg^2+^, the signal in the red channel disappears, while in the green channel it remains stable, reflecting the ratiometric nature of the reaction inside the mitochondria. Without a doubt, the **P-66** is one of the most advanced coumarin probes described in the literature to date.

**Scheme 36:**
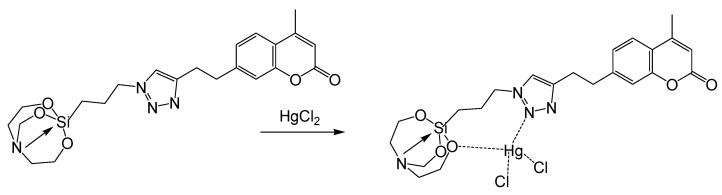
Selective detection of Hg^2+^ with **CS**, **P-67** probe.

In another **CS**, **P-67** probe, coumarin is combined with a rigid silatran system for selective detection of Hg^2+^ [250]. In the presence of Hg^2+^, an immediate spectral shift occurs: the bands at 280 and 252 nm in UV–Vis disappear, leading to an unambiguous absorption signal only for mercury, Scheme 36. In addition, the probe has a very low LOD = 5.4 × 10^−7^ M and high selectivity.

The Hg^2+^ binding mechanism has been confirmed by ^1^H NMR and DFT calculations: Hg^2+^ coordinates simultaneously to the triazole nitrogen atom and the silatran ring oxygen. The probe was tested in real water samples, obtaining a recovery of over 95%, which confirms its practical environmental usefulness. In addition, it displays clear antioxidant activity in the DPPH test (IC_50_ = 9.79 µM), and molecular docking revealed a strong binding ability with the AChE enzyme (ΔG = −8.40 kcal/mol), which significantly exceeds the reference drug galantamine (−7.76 kcal/mol). The results indicate the potential applicability of the probe in the treatment of Alzheimer’s disease.

##### Detection of Other Metals

**Figure 30:**
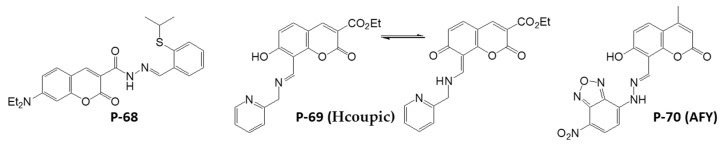
Selected coumarin fluorescent probes for the detection of various metal ions.

Figure 30 shows selected coumarin fluorescent probes designed for the detection of various metal ions (Pd^2+^, Zn^2+^, and Fe^3+^/PPi), illustrating the diversity of their design strategies, response mechanisms, and analytical ranges.

Probe **P-68** is a coumarin derivative equipped with a thioether fragment, exhibiting high selectivity and sensitivity towards Pd^2+^, reaching a low detection threshold of 65 nM due to an effective coordination mechanism [251]. After the analyte binds to the thioether moiety and the Schiff group, there is a strong fluorescence quenching at 495 nm. The probe has been successfully used to image Pd^2+^ in living A549 cells, demonstrating its usefulness in biological and environmental studies.

**P-69**, **HCoupic**, on the other hand, is a tridentate molecule based on a coumarin-pyridyl skeleton, enabling strong, selective enhancement of Zn^2+^ fluorescence (λ_em_ = 438 nm) in HEPES–ACN buffer, ensuring nanomolar detection with clear differentiation from other metal ions [252]. The free probe exhibits a very low fluorescence quantum yield (Φ = 0.0075), which increases significantly to Φ = 0.116 upon Zn^2+^ coordination due to chelation-enhanced fluorescence. The **HCoupic** probe was used to detect Zn^2+^ in both human cells (MDAMB-231 line) and living plant cells (germinating chickpeas), where it allowed visualization of increased fluorescence in response to rising zinc ion concentrations.

The **AFY** (**P-70**)probe is a coumarin linked to a benzofuroxan unit that selectively binds Fe^3+^, causing a significant enhancement of fluorescence and the formation of an **AFY**–Fe^3+^ (1:1) complex, which can then be reversibly deactivated by PPi, enabling sequential detection of Fe^3+^ → PPi [253]. Due to its high sensitivity (LOD: 0.35 µM for Fe^3+^, 0.72 µM for PPi) and biocompatibility, the probe has been used for fluorescent imaging of Fe^3+^ and PPi in vivo in mice and for semi-quantitative detection of Fe^3+^ in real samples.

### 4.4. Subcellular- and Structure-Targeted Probes

Eukaryotic cells contain a number of dedicated organelles such as lysosomes, mitochondria, and lipid droplets (LDs) that play a crucial role in maintaining homeostasis and proper metabolic functions. Although each structure has a distinct function, most complex life processes are carried out through the dynamic cooperation of multiple organelles. An example is lipophagy, during which lysosomes degrade triacylglycerols stored in LDs, providing the cell with high-energy lipids; dysfunction of this process is associated with obesity, diabetes, fatty liver disease, and cancer. Also important are the interactions of LDs and lysosomes with mitochondria, which are involved in the regulation of lipid metabolism, detoxification, and immune signaling. Their malfunction leads to metabolic and neurodegenerative diseases [254,255].

Among the disorders of the central nervous system, Alzheimer’s disease, whose key marker is amyloid β (Aβ) deposits, is of particular importance. Aβ accumulation and aggregation occur at very early stages of the disease, often many years before the onset of clinical symptoms, which is why the ability to monitor the processes of Aβ aggregation, fibrillation, and deposition is currently an important area of diagnostic research [256].

Systemic inflammation caused by infection, chronic disease, or sepsis is associated with elevated cytokine levels and blood–brain barrier dysfunction. One of the most sensitive indicators of inflammatory disorders is a change in mitochondrial viscosity resulting from mitochondrial dysfunction and abnormal inflammasome activation [257,258]. Since mitochondria regulate not only energy production but also apoptosis, metabolism, innate immunity, and signaling, even small changes in their microenvironment can lead to a cascade of disorders affecting the entire body.

In consideration of the above diagnostic challenges, the development of fluorescent probes targeting specific organelles and cellular structures, such as LDs, lysosomes, mitochondria, and pathological Aβ aggregates, is a key tool in modern bioanalytical chemistry. These probes not only enable the detection of specific analytes but, above all, the visualization of dynamic organelle interactions, the monitoring of metabolic changes, and the tracking of pathological processes in vitro and in vivo.

**Figure 31:**
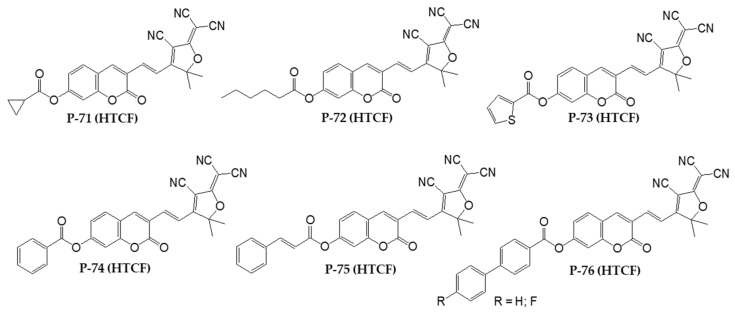
The series of **HTCF** ester probes.

The Ge group designed a series of **HTCF** compounds based on coumarin fluorophore combined with a large hydrophobic ester group, Figure 31 [259]. The large planar shape of the probe and the lack of protonated groups promote natural accumulation in the endoplasmic reticulum (ER).

**Scheme 37:**
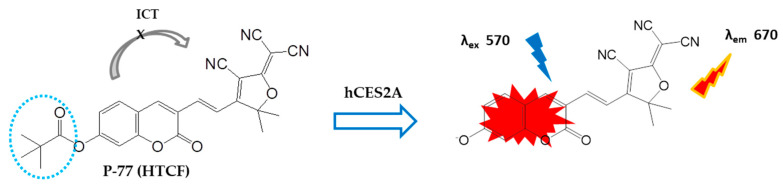
Enzymatic activity of **P-77**.

The series of **HTCF** esters had different structures (straight, branched, and lipophilic alkyl esters). Their hydrolysis by the human Carboxylesterase 2A (hCES2A) enzyme, selectivity towards other esterases, stability, NIR emission after fluorophore release, and accumulation in the ER were evaluated. **P-77 HTCF**, which met all the criteria for enzymatic activity, selectivity, localization, and biological stability, was selected for further study (Scheme 37). The released fluorophore **HTCF** exhibits a moderate fluorescence quantum yield of Φ = 0.055, as determined from spectroscopic measurements.

The mechanism of action of the probe is based on the enzymatic hydrolysis of the ester bond by hCES2A, which unblocks the ICT process and generates an intense OFF–ON signal (more than 60-fold fluorescence enhancement). The probe exhibits excellent selectivity towards hCES2A (76.7-fold advantage over hCES1A), high sensitivity (LOD = 0.021 μg/mL), and stability, enabling quantitative monitoring of enzyme activity in HepG2 cells, liver tissues, and mouse models.

**Scheme 38:**
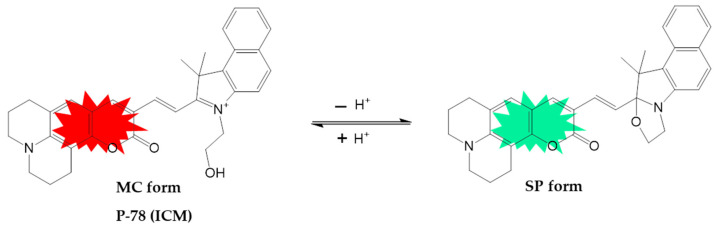
Two reversible forms of **ICM** (**P-78**) probe.

Recently, the Feng group presented **ICM** (**P-78**), which is capable of simultaneously detecting and distinguishing lipid droplets (LDs), lysosomes, and mitochondria in living cells [260]. Its design is based on a hybrid of coumarin and merocyanine, which can exist in two reversible forms: SP (closed, neutral lipophilic form) emitting green light (~480 nm) and preferring LDs, and MC (open, strongly conjugated cationic form) emitting in NIR (~700 nm), which targets mitochondria due to the indolium cation and is formed in acidic lysosomes by protonation of SP, Scheme 38. Differences in the shape, intensity, and location of fluorescence allow mitochondria to be distinguished from lysosomes, despite the same emission wavelength. The probe is highly sensitive (it works even at 1 nM) and enables dynamic tracking of lipophagy and organelle interactions. In addition, it exhibits a high fluorescence quantum yield (Φ = 0.79 in glycerol; Rhodamine B as the reference), consistent with its strong NIR emission.

The unique ICM probe not only enables precise and differential imaging of lipid droplets, mitochondria, and lysosomes, but also allows real-time tracking of lipophagy and dynamic organelle interactions. With reversible structural switching between SP and MC forms, it responds quickly to the microenvironment and enables analysis of mitochondrial stress, lysosomal pH changes, and lipid metabolism disorders. Applications of ICM in living cells demonstrate its unique potential as a tool for studying metabolic pathways and pathologies associated with organelle dysfunction.

**Figure 32:**
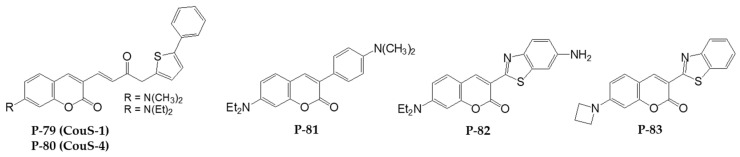
Selected probe for recognition and inhibition of amyloid-β aggregation.

Figure 32 presents the structures of probes that have been designed in recent publications for the selective recognition and inhibition of amyloid-β aggregation.

The **CouS-1** (**P-79**) and **CouS-4** (**P-80**) probes operate in a D-π-A system and exhibit enhanced NIR emission after binding to Aβ (**CouS-1** λ_ex_ ≈ 490–500 nm; λ_em_ ≈ 654–660 nm) **CouS-4** (λ_ex_ ≈ 430–434 nm; λ_em_ ≈ 620–622 nm) penetrate the blood–brain barrier, enabling imaging of amyloid deposits in transgenic mice, and exhibit anti-aggregation activity [261]. Among the CouS series, **CouS-4** exhibits an exceptionally high fluorescence quantum yield (Φ = 0.748 in MeCN), markedly exceeding that of **CouS-1** (Φ = 0.057), highlighting the strong impact of donor–acceptor engineering on emission efficiency in NIR coumarin-based probes. In turn, **P-81-83** probes are coumarin derivatives structurally inspired by Thioflavin-T and differing in donor/acceptor [262]. **P-81** is provided with dimethylaniline as a donor (strong “push–pull”); **P-82** features benzothiazole and an amino group, increasing solubility; **P-83** is a combination of coumarin with benzothiazole and azetidine as an electron donor. After binding to amyloid, the probes exhibit emission in the range of approximately 480–600 nm. **P-81-83** were used to distinguish between pathological and functional amyloids and brain samples from mouse models of Alzheimer’s disease.

**Figure 33:**
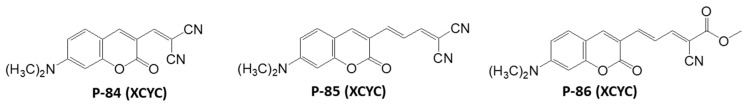
Probes based on a 7-dimethylaminocoumarin skeleton.

The three probes **P-84-86** (**XCYC**) presented by Xu’s group were based on a 7-dimethylaminocoumarin skeleton coupled via a C=C bond system to an electron acceptor containing cyanides or cyanoacrylate derivatives, Figure 33 [263].

Upon binding to Aβ aggregates or changes in the microenvironment (polarization, viscosity), the rotation is restricted and the system becomes rigid, which generates a strong fluorescence enhancement (emission from ~550 nm to 641 nm). Notably, the coumarin-based XCYC probes show high fluorescence quantum yields (Φ = 10.4–36.1% in DMSO), which increase with extended π-conjugation and support their suitability for high-contrast fluorescence imaging. The **P-85** probe, with a strong ICT effect and red-shifted emission (~641 nm), was used for selective detection and monitoring of Aβ aggregation in HT22 cells (mouse hippocampal neuronal cells) and in vivo in transgenic mice.

### 4.5. Emerging Anticancer and Theranostic Coumarin-Based Probes

Theranostic probes with anticancer properties are a rapidly developing direction in medical chemistry and bioimaging. Theranostics combine diagnostic (imaging) and therapeutic functions in a single molecule or nanostructure. This enables simultaneous detection of cancerous changes, real-time monitoring of drug distribution, and local activation of cytotoxic agents within the tumor. Most theranostics use fluorophores emitting in the visible or near-infrared range, ligands directing the probe to overexpressed tumor receptors, or prodrugs activated by tumor biomarkers such as ROS, proteolytic enzymes, reductases, glutathione, or specific metal ions. Increasing attention is also being paid to the possibility of precise imaging of cellular organelles (mitochondria, lysosomes, and nuclei), allowing the tracking of cytotoxicity mechanisms and cellular responses to treatment. Several valuable review articles summarizing the latest advances in the design and application of theranostic probes in oncology have recently been published [264,265,266,267,268,269,270,271,272].

Among theranostic systems, there are also probes based on a coumarin skeleton. Despite intensive development in this area, the number of new, fully functional coumarin-based theranostic probes remains relatively small. This fact is due, among other things, to the challenges associated with the simultaneous integration of a fluorescent module, molecular targeting, and a tumor-activated therapeutic payload into a single small-molecule structure. Nevertheless, emerging publications indicate that coumarins, due to their high photostability, simplicity of synthesis, and the possibility of precise emission tuning, are a promising platform for designing next-generation theranostics. Selected examples of such systems, covering the last three years, are described below.

**Figure 34:**
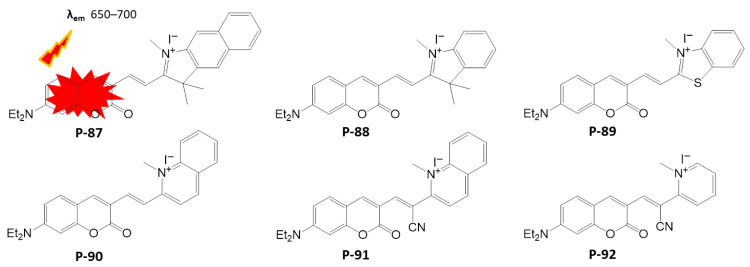
Chemical structures of the coumarin-derived probes designed to target mitochondrial G-quadruplexes.

Lai and Hu designed and synthesized a series of coumarin probes (**P-87**-P-2) differing in substituents modulating affinity for mitochondrial G-quadruplex structures (mtG4 refers to mitochondrial G-quadruplex structures, i.e., non-canonical secondary DNA arrangements formed by guanine-rich sequences within mitochondrial DNA), the ability to localize in mitochondria, and photophysical properties, Figure 34 [273]. After initial screening, the **P-87** structure was selected because it bound strongly to mtG4, had high NIR fluorescence after binding (650–700 nm), and had the desired biological activity.

The **P-87** probe was qualified as a fully functional theranostic probe, and the authors conducted comprehensive biological studies on it: in vitro mitochondrial imaging, 3D spheroid visualization, cell death mechanism analyses (e.g., ROS, apoptosis, autophagy), primarily in HepG2 liver cancer cells. In vivo experiments further confirmed its ability to inhibit tumor growth with low overall toxicity.

**Figure 35:**
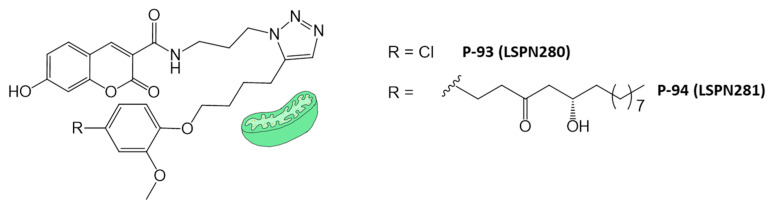
Mitochondria-targeting structures of coumarin hybrids **LSPN280** and **LSPN281**.

The Fuzer’s group presented two hybrid coumarin probes: **LSPN280** (**P-93**) and its modified version, LSPN281 (**P-94**). Both contain a coumarin core linked by a 1,2,3-triazole bond to a bioactive fragment (10-gingerol), but only **LSPN281** exhibited properties corresponding to a theranostic probe, Figure 35 [274].

The **LSPN281** compound was characterized by strong fluorescence (λ_ex_ = 360 nm, λ_em_ = 460 nm), good cellular uptake, and partial localization in mitochondria. Importantly, **LSPN281** showed high cytotoxic activity against MDA-MB-231 TNBC cells (triple-negative breast cancer) with an IC_50_ value of 2.3 μM/48 h. The publication also demonstrates that **LSPN281** induces cytotoxicity by interfering with mitochondrial function, inducing oxidative stress, and activating cell death pathways, which highlights the theranostic nature of this coumarin derivative.

## 5. Conclusions

In recent years, the chemistry of probes based on the coumarin skeleton has been developing exceptionally dynamically, powered by the need for more precise cellular diagnostics and in situ imaging of pathological processes. A review of the available work from 2023–2025 clearly shows that designs based on a simple, easily modifiable coumarin core remain extremely competitive with more complex fluorophores. Their low toxicity, well-known physicochemical properties, and wide-ranging possibilities for donor–acceptor modulation of the π system allow for the creation of probes with high sensitivity and selectivity, while maintaining simple synthesis.

Three trends have been clearly visible in recent years. First, there has been a growing importance of multifunctional probes capable of simultaneously detecting two or more biomarkers (biothiols, ROS/RNS, metabolites, metal ions) or simultaneously detecting chemical and physical parameters (e.g., viscosity, polarization, organelle damage). Molecules such as **BCR**, **TC-2**, and **CP-GSH** perfectly demonstrate that complex disease processes can be successfully tracked in real time using only one fluorophore.

The second direction involves probes emitting in the red and NIR ranges, which provide better tissue penetration and lower autofluorescence levels, significantly improving the quality of in vivo imaging. The use of strong acceptor motifs (malonitrile, dicyanoisophorone, hemicyanine) in conjunction with coumarin allows the signal to be shifted without losing quantum emission efficiency.

The third visible trend is the design of probes targeting specific organelles: mitochondria, ER, lysosomes, lipid droplets. The strategic introduction of anchoring groups (lipophilic cations, indole salts, DNS fragments, highly polarizable D–π–A systems) significantly increases the affinity of probes for specific cellular structures. Recent studies emphasize that organelles do not function in isolation, which is why probes for monitoring interactions between organelles (LD–lysosomes, mitochondria–ER) are becoming particularly important in research on metabolism, inflammation, and neurodegeneration.

Despite significant progress, there are still gaps in the literature that need to be addressed: there are few NIR ratiometric probes that are stable in conditions of strong tissue autofluorescence; validation in large animal models is rarely performed; and there is also a lack of probes that are fully compliant with the principles of green chemistry. Due to their high translational potential, further development should focus on designs with increased photochemical resistance, probes activated in specific disease environments (inflammation), and systems enabling simultaneous diagnosis and therapy (theranostics).

In summary, coumarin probes remain one of the most promising and versatile tools in modern bioanalytics. Their modularity, simplicity of synthesis, and ability to precisely tune optical parameters make them ideally suited to the current needs of molecular diagnostics and cellular imaging. The dynamic development of this class of fluorophores suggests that in the coming years they will become the foundation of many advanced imaging methods and next-generation clinical tools.

## Data Availability

No new data were created or analyzed in this study.
